# Inference of domain-disease associations from domain-protein, protein-disease and disease-disease relationships

**DOI:** 10.1186/s12918-015-0247-y

**Published:** 2016-01-11

**Authors:** Wangshu Zhang, Marcelo P. Coba, Fengzhu Sun

**Affiliations:** Molecular and Computational Biology Program, University of Southern California, 1050 Childs Way, Los Angeles, USA; Zilkha Neurogenetic Institute, Keck School of Medicine, University of Southern California, Los Angeles, CA USA; Department of Psychiatry and Behavioral Sciences, Keck School of Medicine, University of Southern California, Los Angeles, CA USA; Centre for Computational Systems Biology, School of Mathematical Sciences, Fudan University, Shanghai, China

## Abstract

**Background:**

Protein domains can be viewed as portable units of biological function that defines the functional properties of proteins. Therefore, if a protein is associated with a disease, protein domains might also be associated and define disease endophenotypes. However, knowledge about such domain-disease relationships is rarely available. Thus, identification of domains associated with human diseases would greatly improve our understandingof the mechanism of human complex diseases and further improve the prevention, diagnosis and treatment of these diseases.

**Methods:**

Based on phenotypic similarities among diseases, we first group diseases into overlapping modules. We then develop a framework to infer associations between domains and diseases through known relationships between diseases and modules, domains and proteins, as well as proteins and disease modules. Different methods including Association, Maximum likelihood estimation (MLE), Domain-disease pair exclusion analysis (DPEA), Bayesian, and Parsimonious explanation (PE) approaches are developed to predict domain-disease associations.

**Results:**

We demonstrate the effectiveness of all the five approaches via a series of validation experiments, and show the robustness of the MLE, Bayesian and PE approaches to the involved parameters. We also study the effects of disease modularization in inferring novel domain-disease associations. Through validation, the AUC (Area Under the operating characteristic Curve) scores for Bayesian, MLE, DPEA, PE, and Association approaches are 0.86, 0.84, 0.83, 0.83 and 0.79, respectively, indicating the usefulness of these approaches for predicting domain-disease relationships. Finally, we choose the Bayesian approach to infer domains associated with two common diseases, Crohn’s disease and type 2 diabetes.

**Conclusions:**

The Bayesian approach has the best performance for the inference of domain-disease relationships. The predicted landscape between domains and diseases provides a more detailed view about the disease mechanisms.

**Electronic supplementary material:**

The online version of this article (doi:10.1186/s12918-015-0247-y) contains supplementary material, which is available to authorized users.

## Background

Uncovering the mechanisms underlying human complex diseases is one of the central goals of human disease studies. Recent developments in human genetics and computational biology made it possible to identify a number of genes that are associated with complex diseases [1]. For example, recent genome-wide association studies have detected more than 2000 genetic loci associated with human complex diseases or traits [2, 3]. Most of the identified loci, however, represent novel discoveries with no obvious candidate genes and molecular mechanisms [4], rendering difficulties in medical treatment according to genes [5–7]. Even if particular disease associated genes are identified [8–12], narrowing down to particular domains can be challenging because genes may encode for proteins containing a variety of domains. Protein domains are structural units of proteins that can also function independently from other regions of the protein. If a gene product (protein) contains multiple domains [13] and the gene is associated with a disease, one of the domains might be associated with the disease. Narrowing down domains associated with complex diseases will greatly improve our understanding about the pathogenesis of the diseases and facilitate the discovery of drugs as well as personalized medicine.

Several pioneering studies have developed methods for large-scale inference of associations between domains and human diseases based on domain-domain interactions and disease phenotype similarities [14, 15]. These studies have two drawbacks. Firstly, both studies rely on a relatively small set of domain-disease associations compiled by bridging domains that contain known deleterious nsSNPs and human diseases with these nsSNPs [15]. To circumvent this problem we seek evidences of domain-disease associations at the gene level, and instead of considering inadequate number of disease mutations in the domains, we resort to highly abundant publicly available gene-disease associations [16–20]. Secondly, these studies depend on domain-domain interactions that are generally incomplete and contain many false positive and false negative domain interactions [14, 15]. In this study, we use the domain-protein, protein-disease and disease-disease relationships to infer domain-disease relationships without using domain interactions. The basic idea is that if a disease is associated with many genes with their corresponding products (proteins) containing common domains, the common domains are more likely to be associated with the disease.

We *surprisingly* noted that inferring domain-disease relationships based on domain-protein and protein-disease relationship is closely related to the problem of inferring domain-domain interactions based on protein-protein interactions, a problem that have been studied extensively over the past decade [21–32]. Therefore, we adopted some of the promising methods for protein domain interactions based on protein interactions to the inference of domain-disease relationships. These methods include the simple Association method, the Maximum likelihood estimation (MLE) approach studied in Deng et al. [33], Domain pair exclusion analysis (DPEA) approach proposed by Riley et al.[25], a Bayesian version of the MLE approach as developed by Kim et al. [34], and Parsimonious explanation (PE) approach proposed by Guimaraes et al. [26].

Since a particular disease/trait generally has a relatively small number of associated genes and inferring the domains related to the disease based on the small number of proteins can be unreliable, therefore we group single diseases into modules. Each module comprises several disease phenotypes that are highly similar to each other, and different modules may share diseases. This modularization process takes into consideration of the comorbidities of various diseases [35, 36], and overlaps among modules are allowed indicating that one disease may belong to more than one module since for complex disorders, different genetic mutations may lead to the same clinical outcome [37, 38]. For example, schizophrenia is a group of heritable disorders presenting distinct clinical syndromes, and schizophrenia has been shown to be associated with eight separate networks of genetic mutations [20]. Dubowitz syndrome is also a complex disease comprised of multiple, genetically distinct but phenotypically overlapping disorders [39]. As a result, we are able to define a larger number of proteins associated with a particular disease module, and the data insufficiency problem could be solved. In practice we group the diseases based on phenotype similarities obtained from a recent work of Jiang et al. [40] and then identify potentially overlapping disease modules. Then we identify domains associated with each disease contained in these modules.

We develop a framework to infer associations between domains and diseases through known relationships between diseases and modules, domains and proteins, as well as proteins and disease modules. We demonstrate the validity and robustness of these approaches, compare their performance, and predict domain-disease as well as gene-disease associations. We further illustrate the consistency between our inference results and the evidences from genome-wide association studies for two common diseases: Crohn’s disease and type 2 diabetes.

## Materials and methods

### Data sources

The developed scheme to infer domain-disease relationships depends on the relationships between domains and proteins, proteins and diseases, and phenotypic similarities among diseases.

The relationships between domains and human proteins were obtained from the Pfam database, which provides a large collection of both high quality protein domain families (Pfam-A) and low quality protein domain families (Pfam-B) [41]. In version 27.0 of the Pfam-A collection (released in March 2013), there were 146,442 associations between 5561 domains and 100,977 human proteins. A domain is referred as associated with a protein if the protein contains the domain. The relationships between genes and human diseases were extracted from the OMIM database [16], from which 4951 associations between 3313 genes and 4151 diseases were established. Moreover, from a recent work of Jiang et al. [40], we obtained a pair-wise phenotypic similarity profile for 7719 diseases. The author applied text mining techniques to extract feature vectors from three vocabularies, UMLS, MeSH, and HPO, respectively, and a cosine value between two feature vectors for a pair of diseases was calculated to measure their similarity. Here we only selected the phenotype similarities obtained using the UMLS terms, since it has a full coverage of all 7719 disease phenotypes and also achieves the best performance in the leave-one-out cross-validation experiments of measuring the strength of association between a candidate gene and a query disease [40].

### Identification of overlapping disease modules

Given the disease phenotypic similarity profile and a predefined threshold on disease similarity, we construct a disease network with diseases as nodes and two diseases are linked if their pair-wise similarity is above this threshold. Considering computational feasibility, in this paper we choose a threshold of 0.5, and obtain 39,506 linked pairs among 6033 diseases with similarity above this threshold, based on which we construct our disease network.

Then, we use the ClusterONE (Clustering with Overlapping Neighborhood Expansion) method [42] to detect overlapping modules in the disease network. This method was originally proposed to detect potentially overlapping protein complexes from protein-protein interaction data. Here we apply the algorithm to detect overlapping disease modules, with all default parameter values, and retrieve modules containing at least 5 diseases and having *p*-values below 0.05, a measure to evaluate the statistical significance of the module.

**Fig. 1:**
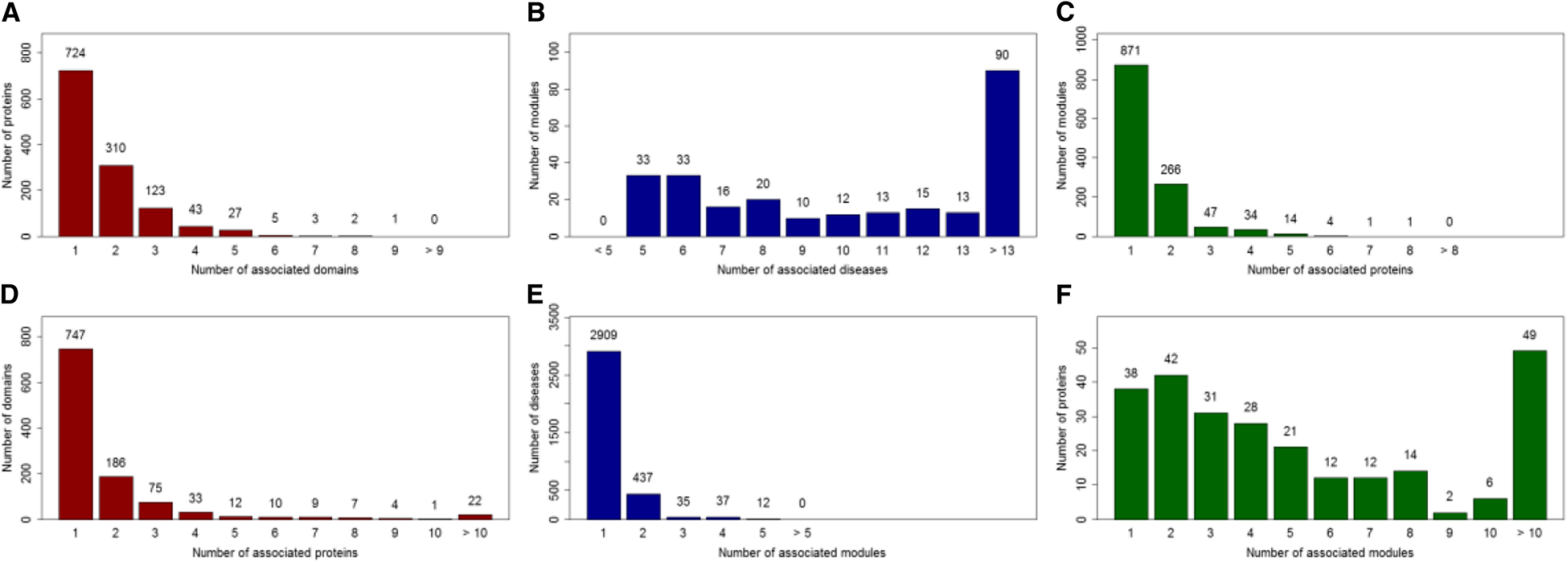
The relationships between the different data types. The histograms of the number of **a** proteins with respect to the number of domains the protein contains, **b** disease modules with respect to the number of diseases the module contains, **c** disease modules with respect to the number of proteins the module associates, **d** domains with respect to the number of proteins the domain associates, **e** diseases with respect to the number of disease modules the disease associates, and **f** proteins with respect to the number of disease modules the protein associates

As a result, we obtain altogether 255 modules, with 3430 diseases included. We say a gene is associated with a module if the gene is associated with at least one of the diseases in the module. We only include modules containing at least one disease having known associated genes. Similarly, we only include genes associated with at least one disease module. A gene is referred as associated with a disease module if it is associated with at least one disease in the module. Finally, we obtain 2096 associations between 1106 domains and 1238 proteins, 4096 associations between 3430 diseases and 255 modules, and 1789 associations between 1238 proteins and 255 modules. Relationships among domains, proteins, diseases and modules are shown in Fig. 1.

As mentioned above we construct our disease network by choosing a threshold of 0.5. Here we also try other thresholds from 0.1 to 0.9 with a step of 0.1 to identify disease modules, and list the numbers of domains, proteins, diseases and modules obtained using the same method, in the Additional file 1: Table S1. From the result we see that when the threshold is selected as 0.5, we obtain the highest number of 255 modules, and when the threshold is selected as 0.4, the numbers of collected domains, diseases and proteins are the largest (1145 domains, 4090 diseases, and 1298 proteins). We also see that as the threshold increases from 0.5 to 0.9, or decreases from 0.4 to 0.1, the numbers of domains, diseases, proteins and modules are all decreasing, but drop relatively slightly when the threshold is between 0.3 and 0.6 (the numbers of domains, diseases, proteins, and modules are larger than 800, 2300, 900, 50, respectively). Since if a threshold larger than 0.6 is selected, there would be fewer diseases that have pair-wise similarities above this threshold, while if a threshold smaller than 0.3 is selected, there would be less disease modules being identified for the increasing insignificance of disease modules due to the excessive connections in the disease network. Therefore, we set the threshold at 0.5 to keep a reasonable number of domains, diseases, proteins and modules.

### Methods to predict domain-disease associations

**Fig. 2:**
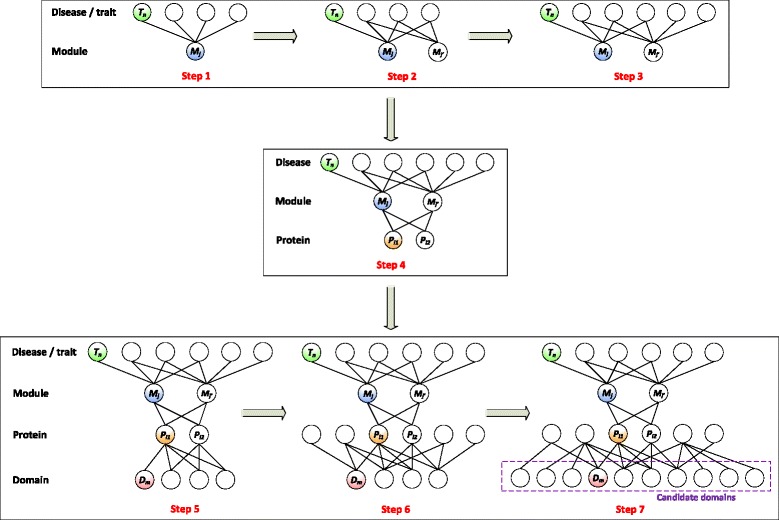
Scheme for predicting domain-disease relationships. Nodes represent diseases/traits, modules, proteins and domains. An edge connecting two nodes represents a known association. Steps 1-7 demonstrate the procedure that, when predicting for a specific disease, how to obtain its candidate domains. Step 1: For a given disease *T*_*n*_, all module(s) containing this disease (in the figure *M*_*j*_) and all the other diseases/traits contained in *M*_*j*_ are extracted. Step 2: Module(s) sharing at least one disease with module *M*_*j*_ (in the figure ${M}_{j^{\prime }}$) are extracted. Step 3: All the other diseases/traits in ${M}_{j^{\prime }}$ are included in the prediction scheme. Step 4: All proteins associated with the set of $\left\{{M}_j,{M}_{j^{\prime }}\right\}$ (in the figure *P*_*i*1_ and *P*_*i*2_) are extracted. Step 5: All domains contained in the set of {*P*_*i*1_, *P*_*i*2_} are included in the prediction scheme. Step 6: All proteins sharing domains with proteins {*P*_*i*1_, *P*_*i*2_} are included in the prediction scheme. Step 7: All the other domains in all proteins produced at Step 6 are included in the prediction scheme and the resulting set of domains are called candidate domains

We develop a scheme to infer domain-disease associations from known relationships between domain-proteins, disease-modules and protein-modules as demonstrated in Fig. 2. As shown in Fig. 2, for a given disease/trait *T*_*n*_, we first extract all module(s) containing this disease (in the figure *M*_*j*_) and all the other diseases contained in *M*_*j*_. Then we consider the modules that share at least one disease with module *M*_*j*_ (in the figure ${M}_{j^{\prime }}$), and also incorporate other diseases in ${M}_{j^{\prime }}$. Next, from the protein-module associations we collect all proteins associated with the set of $\left\{{M}_j,{M}_{j^{\prime }}\right\}$ (in the figure *P*_*i*1_ and *P*_*i*2_), and further find domains contained in these proteins. Similarly, we consider all proteins sharing domains with proteins {*P*_*i*1_, *P*_*i*2_}, and incorporate their domains as well. The resulting set of domains are called candidate domains. Finally, we predict the associations between each of the collected candidate domains and the disease/trait *T*_*n*_. For different diseases, the numbers of candidate domains can vary, and only 247,112 domain-disease relationships will be predicted. For each disease, the number of associated domains is small and thus the standard machine learning based approach cannot be used to predict domain-disease relationships. For clarity of presentation, we summarize all the notations used in the five approaches in Additional file 2.

### The association approach

We first propose a naive Association approach to rank the candidate domain-disease relationship. For a candidate domain *D*_*m*_and a query disease/trait *T*_*n*_, their potential of being associated is measured by the fraction of associated protein-module pairs among all the protein-module pairs containing this domain-disease pair. Specifically, let *A*_*mn*_ = {(*P*, *M*); *D*_*m*_ ∈ *P*, *T*_*n*_ ∈ *M*, *P* is associated with *M*}, where *P* indicates proteins and *M* indicates disease modules, be the set of associated protein-module pairs containing the domain-disease pair (*D*_*m*_, *T*_*n*_). Similarly, let *N*_*mn*_ = {(*P*, *M*); *D*_*m*_ ∈ *P*, *T*_*n*_ ∈ *M*}, be the complete set of protein-module pairs containing (*D*_*m*_, *T*_*n*_). The association score is given by

$$ Score\left({D}_m,{T}_n\right)=\frac{\left|{A}_{mn}\right|}{\left|{N}_{mn}\right|}. $$

If the domain is not included in any proteins that are known to be associated with the disease module containing the disease, the corresponding score is defined as zero [33]. With this definition, we calculate the scores of all candidate domains for each disease.

### The maximum likelihood estimation (MLE) approach

Deng et al. [33] developed a Maximum Likelihood Estimation (MLE) approach to predict domain-domain interactions from protein-protein interactions. Here we extend the approach to infer domain-disease associations, with parameters of this model estimated in terms of the Expectation Maximization (EM) algorithm [33]. Let *D* denote domains, *T* denote diseases/traits, *P* denote proteins and *M* denote disease modules. Let *ϕ*_*mn*_ = 1 denote that domain *D*_*m*_ associates with disease *T*_*n*_ and *ϕ*_*mn*_ = 0, otherwise. Similarly, let *ψ*_*ij*_ = 1 denote protein *P*_*i*_ associates with module *M*_*j*_ and *ψ*_*ij*_ = 0, otherwise.

It is assumed that a protein associates with a module if and only if at least one of domains inside this protein associates with one of diseases belonging to the module. Under this assumption, we have

$$ \Pr\;\left({\psi}_{ij}=1\right)=1-{\displaystyle \prod_{\left({D}_m,{T}_n\right)\in \left({P}_i,{M}_j\right)}\left(1-{\lambda}_{mn}\right)} $$

where *λ*_*mn*_ = Pr (*ϕ*_*mn*_ = 1) denotes the probability that domain *D*_*m*_ associates with disease/trait *T*_*n*_, and we thus define the association score as

$$ Score\left({D}_m,{T}_n\right)={\lambda}_{mn}. $$

If we consider about two types of errors, false positive rate *fp* and false negative rate *fn*, and let *O*_*ij*_ be the indicator variable denoting if protein *P*_*i*_ and module *M*_*j*_ are observed to be associated, then

$$ fp= \Pr \left({O}_{ij}=1\Big|{\psi}_{ij}=0\right) $$

$$ fn= \Pr \left({O}_{ij}=0\Big|{\psi}_{ij}=1\right). $$

Thus, the probability for the observed protein-module association is

Pr(*O*_*ij*_ = 1) = Pr(*ψ*_*ij*_ = 1)(1 − *fn*) + (1 − Pr(*ψ*_*ij*_ = 1))*fp*, and the likelihood function for all the observed protein-module relationships is:

$$ L={\displaystyle \prod_{ij}{\left( \Pr \left({O}_{ij}=1\right)\right)}^{O_{ij}}{\left(1- \Pr \left({O}_{ij}=1\right)\right)}^{1-{O}_{ij}}} $$

The likelihood *L* is a function of *θ* = {*λ*, *fp*, *fn*}. We then apply an EM algorithm to estimate *θ*, as was developed by Deng et al. [33] for the inference of domain-domain interactions. The details of implementing the EM algorithm is described in Additional file 2. We initialize all *λ*_*mn*_^init^ = 1/|*D*^(*n*)^|, where |*D*^(*n*)^| denotes the total number of candidate domains for disease/trait *n*, meaning that each domain has an equal probability to be associated with a specific disease. Also, we perform a grid search for different combinations of {*fp*, *fn*}, and find results are quite robust when *fp* is close to 0 while *fn* is close to 1. Therefore we use {*fp*, *fn*} = {0, 0.9} throughout this paper.

### The domain-disease pair exclusion analysis (DPEA) approach

Riley et al. [25] developed the DPEA (Domain Pair Exclusion Analysis) approach for predicting domain interactions from protein interactions. Here we extend the approach to predict domain-disease associations based on protein-module relationships. This approach extends the MLE approach, by first estimating the probability for each potential domain-disease association through the MLE approach, and then measuring association strength in terms of the changes in likelihood ratios when a potential underlying domain-disease pair is assumed to be not associated [25].

The DPEA approach does not consider the false positive and negative rates inherent in protein-module associations, which means, {*fp*, *fn*} = {0, 0}. However, it identifies low-probable but high-specificity domain-disease associations that are not detected by the MLE approach.

The likelihood *L* is a function of *θ* = *λ*, and we apply an EM algorithm to estimate *θ*. Then we use the log-likelihood ratio without restricting *λ*_*mn*_ versus that when *λ*_*mn*_ = 0 to measure the strength of association between the domain *D*_*m*_ and trait *T*_*n*_. The score is thus defined as

$$ \begin{array}{l} Score\left({D}_m,{T}_n\right)={\displaystyle \sum_{ij} \log \frac{ \Pr \left({O}_{ij}=1\Big|{D}_m,{T}_n\kern0.5em \mathrm{can}\kern0.5em \mathrm{associate}\right)}{ \Pr \left({O}_{ij}=1\Big|{D}_m,{T}_n\kern0.5em \mathrm{do}\ \mathrm{not}\kern0.5em \mathrm{associate}\right)}}\\ {}\kern1.5em ={\displaystyle \sum_{ij} \log \frac{1-{\displaystyle \prod_{\left({D}_k,{T}_l\right)\in \left({P}_i,{M}_j\right)}\left(1-{\lambda}_{kl}\right)}}{1-{\displaystyle \prod_{\left({D}_k,{T}_l\right)\in \left({P}_i,{M}_j\right)}\left(1-{\overline{\lambda}}_{kl}^{mn}\right)}}}\end{array} $$

where ${\overline{\lambda}}^{mn}$ is obtained from *λ* by setting the probability of domain *D*_*m*_ associated with disease *T*_*n*_ to be zero, and is also estimated by the EM algorithm. The details of deducing the score function of the DPEA approach is described in Additional file 2.

### The Bayesian approach

Kim et al. [34] extended the MLE approach to a Bayesian approach for the prediction of domain-domain interactions based on protein interactions. We adopt the Bayesian approach for the prediction of domain-disease associations based on protein-module associations. We assume a uniform prior distributions of false positive rate *fp* and false negative rate *fn*: *fp* ~ Unif[*u*_*p*_, *v*_*p*_] and *fn* ~ Unif[*u*_*n*_, *v*_*n*_], and also assume the domain-disease association probability *λ*_*mn*_ has a Beta prior distribution: *λ*_*mn*_ ~ Beta(*α*, *β*) as in Kim et al. [34]. Therefore, the posterior distribution of *λ*_*mn*_ is proportional to

$$ \begin{array}{l}\left[\lambda \Big| rest\right]\propto L\left(O\Big|fn,fp,\lambda \right)f\left(\lambda \Big|fn,fp\right)\\ {}\kern4em \propto {\displaystyle \prod_{ij}{\left( \Pr \left({O}_{ij}=1\right)\right)}^{O_{ij}}{\left(1- \Pr \left({O}_{ij}=1\right)\right)}^{1-{O}_{ij}}}\times f\left(\lambda \Big|fn,fp\right)\\ {}\kern4em \propto {\displaystyle \prod_{ij}{\left[{h}_{ij}\left(\lambda \right)\left(1-fn\right)+\left\{1-{h}_{ij}\left(\lambda \right)\right\}fp\right]}^{O_{ij}}{\left[1-{h}_{ij}\left(\lambda \right)\left(1-fn\right)-\left\{1-{h}_{ij}\left(\lambda \right)\right\}fp\right]}^{1-{O}_{ij}}}\\ {}\kern4em \times f\left(\lambda \Big|fn,fp\right)\end{array} $$

Here *λ* = {*λ*_*mn*_; *D*_*m*_ ∈ *P*, *T*_*n*_ ∈ *M*}, where *P* indicates proteins and *M* indicates disease modules, and *h*_*ij*_(*λ*) = Pr (*ψ*_*ij*_ = 1).

In addition, the posterior distributions of *fp* and *fn* are proportional to

$$ \begin{array}{l}\left[fp\Big| rest\right]\propto L\left(O\Big|fn,fp,\lambda \right)f\left(fp\Big|\lambda, fn\right)f\left(fn\Big|\lambda \right)\\ {}\kern4em \propto {\displaystyle \prod_{ij}{\left( \Pr \left({O}_{ij}=1\right)\right)}^{O_{ij}}{\left(1- \Pr \left({O}_{ij}=1\right)\right)}^{1-{O}_{ij}}}\times f\left(fp\Big|\lambda, fn\right)f\left(fn\Big|\lambda \right)\\ {}\kern4em \propto {\displaystyle \prod_{ij}{\left( \Pr \left({O}_{ij}=1\right)\right)}^{O_{ij}}{\left(1- \Pr \left({O}_{ij}=1\right)\right)}^{1-{O}_{ij}}}\times f\left(fp\Big|\lambda, fn\right)\\ {}\kern4em \propto {\displaystyle \prod_{ij}{\left[{h}_{ij}\left(\lambda \right)\left(1-fn\right)+\left\{1-{h}_{ij}\left(\lambda \right)\right\}fp\right]}^{O_{ij}}{\left[1-{h}_{ij}\left(\lambda \right)\left(1-fn\right)-\left\{1-{h}_{ij}\left(\lambda \right)\right\}fp\right]}^{1-{O}_{ij}}}\\ {}\kern4em \times f\left(fp\Big|\lambda, fn\right)\end{array} $$

and

$$ \begin{array}{l}\left[fn\Big| rest\right]\propto L\left(O\Big|fn,fp,\lambda \right)f\left(fn\Big|\lambda, fp\right)f\left(fp\Big|\lambda \right)\\ {}\kern4em \propto {\displaystyle \prod_{ij}{\left( \Pr \left({O}_{ij}=1\right)\right)}^{O_{ij}}{\left(1- \Pr \left({O}_{ij}=1\right)\right)}^{1-{O}_{ij}}}\times f\left(fn\Big|\lambda, fp\right)f\left(fp\Big|\lambda \right)\\ {}\kern4em \propto {\displaystyle \prod_{ij}{\left( \Pr \left({O}_{ij}=1\right)\right)}^{O_{ij}}{\left(1- \Pr \left({O}_{ij}=1\right)\right)}^{1-{O}_{ij}}}\times f\left(fn\Big|\lambda, fp\right)\\ {}\kern4em \propto {\displaystyle \prod_{ij}{\left[{h}_{ij}\left(\lambda \right)\left(1-fn\right)+\left\{1-{h}_{ij}\left(\lambda \right)\right\}fp\right]}^{O_{ij}}{\left[1-{h}_{ij}\left(\lambda \right)\left(1-fn\right)-\left\{1-{h}_{ij}\left(\lambda \right)\right\}fp\right]}^{1-{O}_{ij}}}\\ {}\kern4em \times f\left(fn\Big|\lambda, fp\right)\end{array} $$

An adaptive rejection sampling algorithm [43] is applied to sample from the posteriors, and parameters can be estimated using the posterior means.

### The parsimony explanation (PE) approach

Guimaraes et al.[26] originally developed a PE approach for predicting domain interactions based on protein interactions. Here we extend the PE approach for predicting domain-disease associations from protein-module associations. The PE approach formulates the problem of predicting domain-disease associations into a linear programming framework. The optimization objective is to minimize the number of overall domain-disease associations necessary to justify each of the underlying protein-module associations. We define a set of domain-disease pairs *Δ* = {{*D*_*m*_, *T*_*n*_}|*D*_*m*_ ∈ *P*, *T*_*n*_ ∈ *M*, and *P* associates with *M*}, where *P* indicates proteins and *M* indicates disease modules, underlying all associated protein-module pairs. Therefore, domain *D*_*m*_ and disease *T*_*n*_ are assumed to have an association variable *x*_*mn*_ if and only if there exists an associated protein-module pair *P* and *M* containing domain *D*_*m*_ and disease *T*_*n*_, respectively. We propose to solve the following linear programming (LP) problem:

Minimize $$ {\displaystyle \sum_{\left\{{D}_m,{T}_n\right\}\in \varDelta }{x}_{mn}}, $$

Subject to: ${\displaystyle \sum_{\left({D}_m,{T}_n\right)\kern0.5em \in \kern0.5em \left({P}_i,{M}_j\right)}{x}_{mn}}\ge 1$, for every associated protein-module pair (*P*_*i*_, *M*_*j*_) corresponding to one linear constraint. The resulting value *x*_*mn*_ is then used to measure the strength of potential domain-disease association between domain *D*_*m*_ and disease *T*_*n*_. To account for the noise in the protein-module associations, we define *r* as the reliability rate, which is, the probability that a protein-module association actually exists. For a pre-selected *r*, we include each protein-module association into the constraints with probability *r*, and perform the linear programming 1000 times. The average *x*_*mn*_ is defined as the LP-score and used to measure the strength of association between domain *D*_*m*_ and disease *T*_*n*_.

To control for possible over-prediction of associations between frequently occurring domain-disease pairs, we assign a promiscuity versus witnesses (pw)-score to each domain-disease pair. Specifically, a pw-score between domain *D*_*m*_ and disease *T*_*n*_ is defined as

$$ \mathrm{p}\mathrm{w}\hbox{-} \mathrm{score}\left({D}_m,{T}_n\right)= \min \left(p\hbox{-} \mathrm{value}\left({D}_m,{T}_n\right),\kern0.5em {\left(1-r\right)}^{w\left({D}_m,{T}_n\right)}\right) $$

where *w*(*D*_*m*_, *T*_*n*_) is the number of occurrences (witnesses) for a given domain-disease pair (*D*_*m*_, *T*_*n*_) in each associated protein-module pair (*P*_*i*_, *M*_*j*_), and *r* is the reliability rate of the protein-module association. Thus, the value of ${\left(1-r\right)}^{w\left({D}_m,{T}_n\right)}$ is the probability that all witnesses of domain-disease pairs in (*P*_*i*_, *M*_*j*_) are false positives. We then define *p* ‐ value(*D*_*m*_, *T*_*n*_) to measure the influence of the frequently occurring domain-disease pairs on the LP-score. To estimate this, we generate 1000 random protein-module associations by preserving the same protein-domain and module-disease compositions as well as the total number of protein-module associations, but selecting associations between proteins and modules at random. We perform the linear programming 1000 times, and the *p* ‐ value(*D*_*m*_, *T*_*n*_) is calculated as the frequency of obtaining the same or higher LP-score in the 1000 runs when the protein-module pair containing the domain-disease pair (*D*_*m*_, *T*_*n*_) is randomized.

Potential associations with LP-scores above a threshold and pw-scores below another threshold are predicted to be putative associations between domains and diseases.

### Validation methods and evaluation criteria

**Fig. 3:**
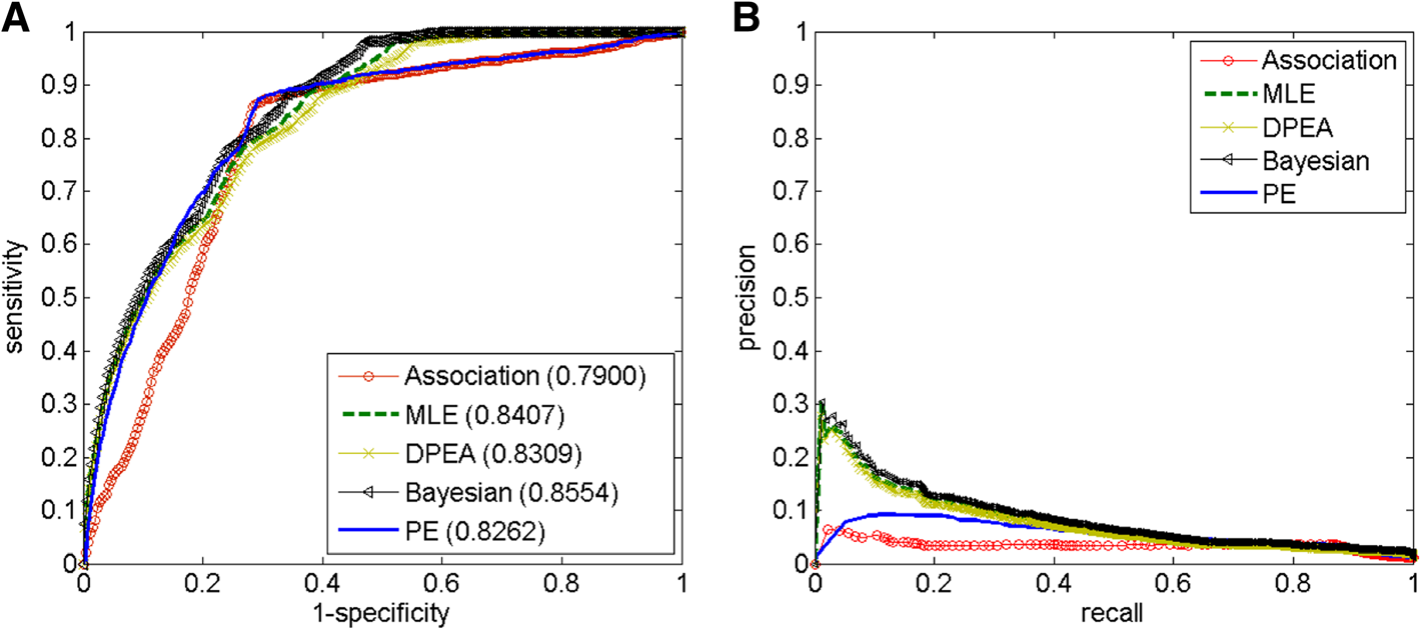
Receiver Operating Characteristic (ROC) and Precision-Recall curves of the different approaches. The figure shows ROC curves (Subplot **a**) and precision-recall curves (Subplot **b**) of the Association, MLE (*fp* = 0, *fn* = 0.9), DPEA, Bayesian (*u*_*p*_, *u*_*n*_ = 0, *v*_*p*_, *v*_*n*_ = 1, and *α* = 2, *β* = 2), and PE (*r* = 100 %, and pw threshold ≤ 0.01) approaches, respectively. Based on both ROC and precision-recall curves, the three MLE based approaches including DPEA, MLE and Bayesian outperform PE and Association. The Bayesian approach performs slightly better than DPEA and MLE

We compile an independent validation set to test how well the Association, MLE, DPEA, Bayesian, and PE approaches perform in recovering known associations between domains and diseases obtained from the Ensembl database (Human Release 80, accessed in June 2015) [44]. By running the BioMart tool [45] within Ensembl, we collect 3004 associations between 869 domains and 1484 diseases in our study. We then consider these 3004 associations as positives, and all the other 244,108 possible domain-disease associations as negatives (247,112 candidate domain-disease associations minus 3004 known associations). For each of the approaches developed above, we rank the domain-disease pairs together in descending order of their scores. Given a predefined threshold of rank, we define the *sensitivity* (or called *recall*) as the percentage of positive domain-disease pairs that are ranked above the threshold, the *specificity* as the percentage of negative domain-disease pairs that are ranked below the threshold, and the *precision* as the percentage of domain-disease pairs ranked above the threshold that are truly positives. By varying the threshold values, we are able to plot a ROC (*R*eceiver *O*perating *C*haracteristic) curve, which demonstrates the relationships between *sensitivity* and 1-*specificity*, as shown in Fig. 3 (a), and a Precision-Recall curve, which demonstrates the relationships between *precision* and *recall*, as shown in Fig. 3 (b).

We use three criteria to measure the performance of each approach. The first criterion is the *Area Under the ROC curve* (*AUC*), which provides an overall measure for the performance of the prioritization approach. The second criterion is the *Accuracy*, which is the fraction of positive domain-disease pairs with the corresponding domain being ranked at the top 10 of the disease. Since the average length of candidate domain lists for all 3430 diseases is 72 (247,112 candidate domain-disease associations divided by 3430), therefore by a random guess the probability to rank a known domain-disease association at the top 10 is 10/72 ≈ 0.1389. Obviously, a high value of accuracy that is much larger than 0.1389 represents an approach with high prediction power. The third criterion is the *Mean Rank Ratio (MRR)*. For each positive domain-disease pair, the rank ratio is calculated as the rank of corresponding domain for the disease divided by the total number of candidate domains, and the mean rank ratio is the average of rank ratios for all the positive associations in the validation experiment. This criterion provides a summary of the ranks of all positive domain-disease associations, and the smaller the mean rank ratio is, the better the approach performs.

## Results

### Validation and comparison of different approaches

On the basis of the compiled known associations between domains and diseases that comprise an independent test data set, we implement a series of large-scale validation experiments to demonstrate the effectiveness of all the approaches developed in this paper. As is described in the “Materials and Methods” section, in each run of the experiments, we prioritize candidate domains according to the scores generated by a selected approach under investigation, with performances being evaluated by AUC, accuracy and mean rank ratio of known domain-disease associations, and list the results in Table 1. Corresponding ROC curves together with the Precision-recall curves of the Association, MLE, DPEA, Bayesian, and PE approaches are shown in Fig. 3.

**Table 1:** Performance of the five approaches

Approach	AUC	Accuracy	Mean rank ratio
Association	0.7900	0.6256	0.2432
MLE	0.8407	0.7074	0.1914
DPEA	0.8309	0.6513	0.2177
Bayesian	0.8554	0.7289	0.1872
PE	0.8262	0.6525	0.2282

The AUC, accuracy and mean rank ratio of the Association, MLE (*fp* = 0, *fn* = 0.9), DPEA, Bayesian (*u*_*p*_, *u*_*n*_ = 0, *v*_*p*_, *v*_*n*_ = 1, and *α* = 2, *β* = 2), and PE (*r* = 100 %, and pw threshold ≤ 0.01) approaches for predicting domain-disease associations, respectively

From the results in Table 1 we see that, all the approaches perform reasonably well recovering the known associations between domains and diseases. The AUC scores obtained using the five approaches are all above 0.79, the accuracies are all above 0.62, and the mean rank ratios are all below 0.25, suggesting the effectiveness of the approaches. The best performance is achieved using the Bayesian approach, with an AUC score of 0.8554, accuracy of 0.7289 and a mean rank ratio of 0.1872. The MLE approach is a little inferior to the Bayesian approach whose AUC score is 0.8407, accuracy is 0.7074, and the mean rank ratio is 0.1914. The DPEA and PE approaches are two less effective ones with the performances being quite close to each other (AUCs are 0.8309 and 0.8262, accuracies are 0.6513 and 0.6525, and mean rank ratios are 0.2177 and 0.2282), while the Association approach performs worst with a AUC score of 0.7900, accuracy of 0.6256, and a mean rank ratio of 0.2432. From Fig. 3, we observe that the ROC curve of the Bayesian approach is above those of the other approaches, suggesting that the performance of Bayesian approach is superior over that of the others. From the precision-recall curves we also see the same trend. Therefore, we see from the results that the predictive power of the five approaches follows an order of: Bayesian > MLE > DPEA > PE > Association.

### Robustness of the approaches

We notice that some of these approaches contain free parameters such as the false positive rate *fp* and the false negative rate *fn* in the MLE approach, the hyper-parameters in prior distributions of the Bayesian approach, in addition to the reliability rate *r* and the pw-score threshold in the PE approach. In the above validation experiments, we only use predefined values of these parameters for simplicity, yet there is still necessity to show whether these approaches are sensitive to these parameters. Hence, for each approach we select several values across the range of corresponding parameters, perform the validation experiments, and see how the results change accordingly.

**Fig. 4:**
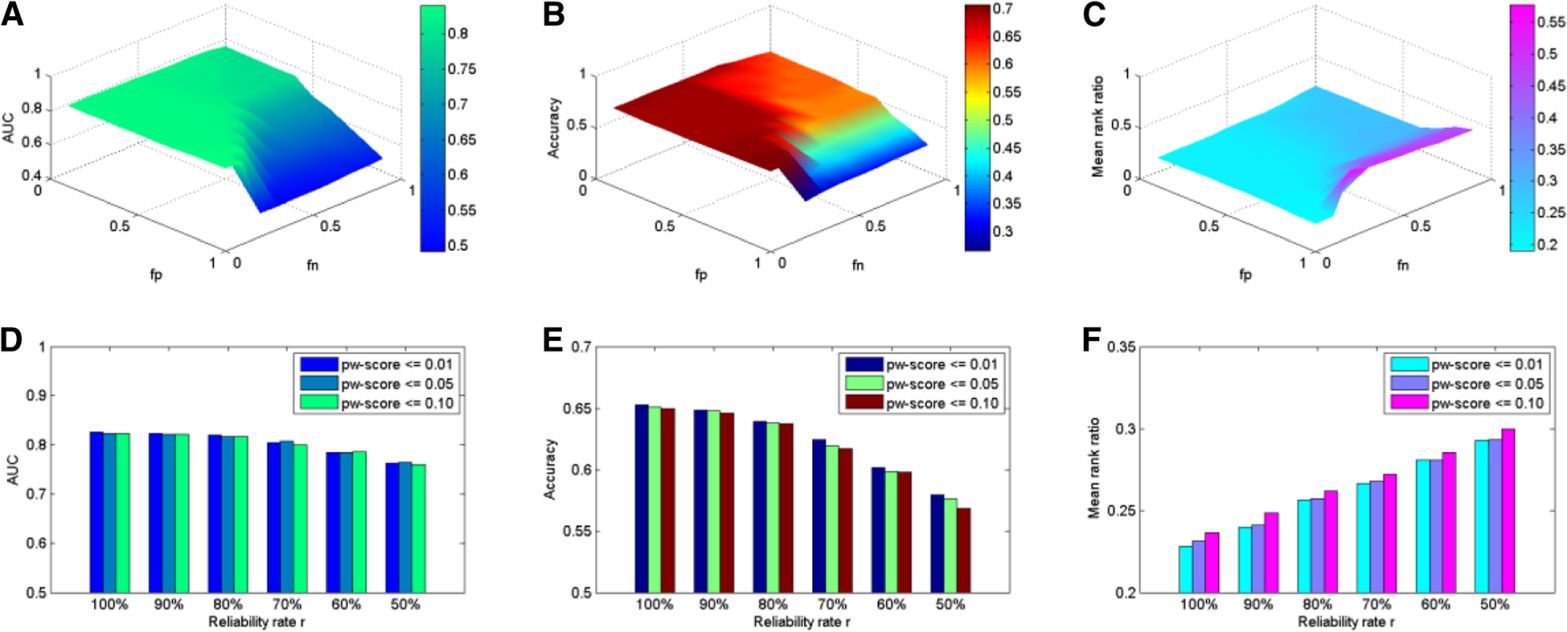
Influences of the free parameters on the performance of the MLE and PE approaches. Horizontally, Subplots **a**-**c** illustrate the influences of false positive rate (*fp*) and false negative rate (*fn*) on AUC, accuracy and the mean rank ratio of the of the MLE approach; Subplots **d**-**f** illustrate the influences of reliable rate (*r*) and pw threshold on AUC, accuracy and the mean rank ratio of the PE approach. Vertically, Subplots **a** and **d** illustrate AUC scores; Subplots B and E illustrate accuracies; Subplots C and F illustrate mean rank ratios, respectively

Specifically, for the MLE approach we implement a grid search of *fp* and *fn* at the same time, increasing from 0 to 1 with a step of 0.1, respectively. Performances in terms of AUC, accuracy, and mean rank ratio are shown in Fig. 4 (a-c) and Additional file 1: Table S2. From the figure we can see when both *fp* and *fn* are relatively large (the shading areas in Additional file 1: Table S2), the AUC is lower than 0.72, the accuracy is lower than 0.48, and the mean rank ratio is higher than 0.35. Otherwise as *fp* increases, performances become worse. For example, when *fn* = 0.2, the peak performance is obtained at *fp* = 0 (AUC = 0.8313, accuracy = 0.6935, and mean rank ratio = 0.2128), and the worst performance is obtained at *fp* = 0.9 (AUC = 0.7944, accuracy = 0.6069, and mean rank ratio = 0.2815). The best performance is obtained at *fp* = 0 combined with *fn* = 0.9 (AUC = 0.8407, accuracy = 0.7074, and mean rank ratio = 0.1914). From the results we conjecture that better performance is usually obtained when *fp* is small and *fn* is large. This is reasonable since according to the experience of Deng et al. [33], *fp* usually takes a very small value while *fn* usually takes a large value close to 1. In the further analysis we just use *fp* = 0 and *fn* = 0.9 so as to acquire a higher predictive power.

Additionally, for the Bayesian approach, we have assigned a beta prior distribution for *λ*, and to eliminate the effect of shape hyper-parameters on the results, we choose to use a 0-1 uniform distribution instead. After re-run of the validation experiments, we obtain AUC = 0.8553, accuracy = 0.7289, and mean rank ratio = 0.1874, which brings tiny change to the inference results. Hence we conjecture that the Bayesian approach is quite robust when an appropriate flat prior distribution is used, and compared to the likelihood functions, priors are not factors playing decisive roles here.

Finally, for the PE approach we decrease the reliability rate *r* from 100 to 50 % with a step of 10 % while fixing the pw-score threshold as 0.01, 0.05, and 0.10, respectively, with the changes of AUC, accuracies and mean rank ratios shown in Fig. 4 (d-f) and Additional file 1: Table S3. First, as the pw-score threshold increases, performances in terms of the three criteria become worse. For example when *r* is 100 %, the performance obtained at pw threshold = 0.01 is the best (AUC = 0.8262, accuracy = 0.6525, and mean rank ratio = 0.2282), followed by the performance obtained at pw threshold = 0.05 (AUC = 0.8238, accuracy = 0.6509, and mean rank ratio = 0.2314), while the worst performance is obtained at pw threshold = 0.10 (AUC = 0.8235, accuracy = 0.6499, and mean rank ratio = 0.2365). The same trend can also be observed for other values of *r*. Second, as *r* decreases, performances become worse since both AUC and accuracy increase, and mean rank ratio decreases. For example, when the pw-score threshold is 0.01, the peak performance is obtained at *r* = 100 % (AUC = 0.8262, accuracy = 0.6525, and mean rank ratio = 0.2282), and the worst performance is obtained at *r* = 50 % (AUC = 0.7635, accuracy = 0.5793, and mean rank ratio = 0.2929). The change of AUC is not as obvious as the other two criteria, and all the values for the three criteria are generally stable. We then conjecture that the PE approach is not sensitive to this free parameter on the basis that pw threshold is smaller than 0.10 and *r* is greater than 50 %. In the further analysis we use pw threshold = 0.01 and *r* = 100 % to achieve maximal number of new predictions according to Guimaraes et al. [26].

### Effects of disease modularization on the performance of different approaches

**Fig. 5:**
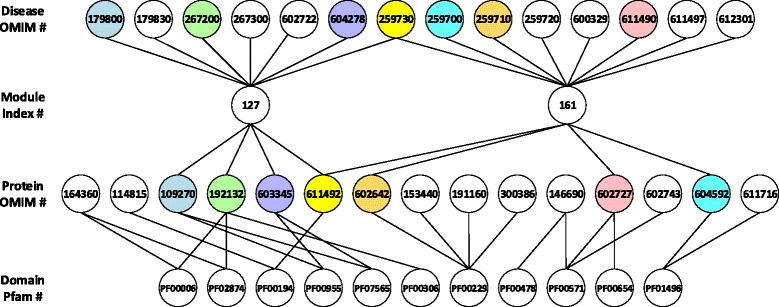
Example for illustration of module effect. Nodes represent diseases with OMIM numbers, modules with index numbers, proteins with OMIM numbers and domains with Pfam numbers. Edges connecting two nodes represent a known association. Nodes with the same background colors represent 7 known associations between corresponding diseases and proteins. (i) Disease OMIM # corresponds to disease/trait names as: [179800]: RENAL TUBULAR ACIDOSIS, DISTAL, AUTOSOMAL DOMINANT. [179830]: RENAL TUBULAR ACIDOSIS, PROXIMAL. [267200]: RENAL TUBULAR ACIDOSIS III. [267300]: RENAL TUBULAR ACIDOSIS, DISTAL, WITH PROGRESSIVE NERVE DEAFNESS. [602722]: RENAL TUBULAR ACIDOSIS, DISTAL, AUTOSOMAL RECESSIVE; RTADR. [604278]: RENAL TUBULAR ACIDOSIS, PROXIMAL, WITH OCULAR ABNORMALITIES AND MENTAL RETARDATION. [259730]: OSTEOPETROSIS, AUTOSOMAL RECESSIVE 3; OPTB3. [259700]: OSTEOPETROSIS, AUTOSOMAL RECESSIVE 1; OPTB1. [259710]: OSTEOPETROSIS, AUTOSOMAL RECESSIVE 2; OPTB2. [259720]: OSTEOPETROSIS, AUTOSOMAL RECESSIVE 5; OPTB5. [600329]: OSTEOPETROSIS AND INFANTILE NEUROAXONAL DYSTROPHY. [611490]: OSTEOPETROSIS, AUTOSOMAL RECESSIVE 4; OPTB4. [611497]: OSTEOPETROSIS, AUTOSOMAL RECESSIVE 6; OPTB6. [612301]: OSTEOPETROSIS, AUTOSOMAL RECESSIVE 7; OPTB7. (ii) Protein OMIM # corresponds to gene name as: [164360]: ATP5A1; [114815]: CA8; [109270]: SLC4A1; [192132]: ATP6V1B1; [603345]: SLC4A4; [611492]: CA2; [602642]: TNFSF11; [153440]: LTA; [191160]: TNF; [300386]: CD40LG; [146690]: IMPDH1; [602727]: CLCN7; [602743]: PRKAG2; [604592]: TCIRG1; [611716]: ATP6V0A2. (iii) Domain Pfam # corresponds to domain name as: [PF00006]: ATP-synt_ab; [PF02874]: ATP-synt_ab_N; [PF00194]: Carb_anhydrase; [PF00955]: HCO3_cotransp; [PF07565]: Band_3_cyto; [PF00306]: ATP-synt_ab_C; [PF00229]: TNF; [PF00478]: IMPDH; [PF00571]: CBS; [PF00654]: Voltage_CLC; [PF01496]: V_ATPase_I

Beyond the parameters in the approaches, our proposed inference scheme also highly relies on the formulation of disease modules. To demonstrate it, we select the disease RENAL TUBULAR ACIDOSIS DISTAL AUTOSOMAL DOMINANT (OMIM: 179800) as an example. Using the scheme illustrated in Fig. 2, we extract all relevant relationships among modules, diseases, proteins and domains, as shown in Fig. 5, to infer the strength of associations among all domains and diseases shown in the figure. Each disease and its known associated gene (protein) are marked with the same color.

By clustering single disease phenotypes into modules in a disease network, known associations between proteins and diseases are actually expanded. Considering a disease that has no known associated proteins, if this disease belongs to a module whose other member diseases have known associated proteins, then as a result, relations between these proteins and the disease are actually bridged, the hidden effects of which would greatly benefit us to uncover more novel associated domains or genes for the disease. For example, the disease RENAL TUBULAR ACIDOSIS PROXIMAL (OMIM: 179830) does not have any known associated genes in the OMIM database. However, since it belongs to the same module as the disease RENAL TUBULAR ACIDOSIS DISTAL AUTOSOMAL DOMINANT (OMIM: 179800), we are able to predict its associated domains. We further map the predicted domains to all genes with corresponding proteins containing these domains, and therefore we are able to predict the genes associated with the disease.

Moreover, we compare the disease RENAL TUBULAR ACIDOSIS DISTAL AUTOSOMAL DOMINANT (OMIM: 179800) with another disease OSTEOPETROSIS, AUTOSOMAL RECESSIVE 3 (OPTB3; OMIM: 259730), since the former disease is only contained in module #127 and the latter one is contained in both module #127 and module #161. The results in terms of all five approaches are listed in Table 2. First, we observe that the Association and PE approaches generate zero scores for a domain-disease association if the domain is not contained in any proteins that are associated with the modules containing the disease, while the other three approaches usually provide such association with a non-zero score. This explains why the Association and the PE approach have relatively lower predictive power compared to the other three approaches. Second, if we only focus on disease RENAL TUBULAR ACIDOSIS DISTAL AUTOSOMAL DOMINANT (OMIM: 179800), we observe that comparatively the domains ATP-synt_ab (PF00006), ATP-synt_ab_N (PF02874), Carb_anhydrase (PF00194), HCO3_cotransp (PF00955), Band_3_cyto (PF07565) and ATP-synt_ab_C (PF00306) have more indirect associations with the disease, compared to the domains TNF (PF00229), IMPDH (PF00478), CBS (PF00571), Voltage_CLC (PF00654), and V_ATPase_I (PF01496), and therefore should have comparatively higher scores. This is generally the case when we see from the left half part of Table 2, except for the results of DPEA method, that due to the too many connection with proteins, the domain PF00229 has the highest score. Third, if focus on domains ATP-synt_ab (PF00006), ATP-synt_ab_N (PF02874), Carb_anhydrase (PF00194), HCO3_cotransp (PF00955), Band_3_cyto (PF07565) and ATP-synt_ab_C (PF00306), we observe that their scores obtained for disease RENAL TUBULAR ACIDOSIS DISTAL AUTOSOMAL DOMINANT (OMIM: 179800) are generally higher than those obtained for disease OSTEOPETROSIS, AUTOSOMAL RECESSIVE 3 (OPTB3; OMIM: 259730). Since the disease RENAL TUBULAR ACIDOSIS DISTAL AUTOSOMAL DOMINANT (OMIM: 179800) is only contained in one module while disease OSTEOPETROSIS, AUTOSOMAL RECESSIVE 3 (OPTB3; OMIM: 259730) is contained in two, we conjecture from the results that, for a domain under investigation, if a disease is only contained in one module associated with the proteins containing this domain while another disease is also contained in other modules that are not associated with the corresponding proteins, usually the first disease would receive higher score to be associated with this domain since its relationship with the domain is more “specific”.

**Table 2:** Effect of disease modularization on the performances of different approaches

		Disease (OMIM ID)									
		RENAL TUBULAR ACIDOSIS, DISTAL, AUTOSOMAL DOMINANT (OMIM: 179800)					OSTEOPETROSIS, AUTOSOMAL RECESSIVE 3; OPTB3 (OMIM: 259730)				
		Association	MLE	DPEA	Bayesian	PE	Association	MLE	DPEA	Bayesian	PE
Domain (Pfam ID)	ATP-synt_ab (PF00006)	0.5	0.0971	0.0041	0.6256	0.0476	0.25	0.0930	0.0024	0.6084	0.0476
	ATP-synt_ab_N (PF02874)	0.5	0.0971	0.0041	0.6843	0.0476	0.25	0.0930	0.0024	0.6366	0.0476
	Carb_anhydrase (PF00194)	0.5	0.1566	0.062	0.8031	0.1646	0.5	0.1499	0.0541	0.7741	0.1210
	HCO3_cotransp (PF00955)	1	0.1300	0.0065	0.7569	0.0714	0.5	0.1086	0.0003	0.6438	0.0714
	Band_3_cyto (PF07565)	1	0.1300	0.0065	0.7078	0.0714	0.5	0.1086	0.0003	0.6859	0.0714
	ATP-synt_ab_C (PF00306)	1	0.1069	0.0002	0.6879	0.0476	0.5	0.0983	0.0001	0.6048	0.0476
	TNF (PF00229)	0	0.0806	0.1900	0.4526	0	0.125	0.0968	0.1906	0.5360	0.1250
	IMPDH (PF00478)	0	0.0874	0.007	0.4572	0	0	0.0878	0.0059	0.4301	0
	CBS (PF00571)	0	0.0851	0.0676	0.4381	0	0.1667	0.0910	0.0646	0.6035	0.06250
	Voltage_CLC (PF00654)	0	0.0874	0.0058	0.4547	0	0.5	0.1040	0.0061	0.6864	0.06250
	V_ATPase_I (PF01496)	0	0.0808	0.0823	0.4801	0	0.25	0.1103	0.0823	0.7057	0.1250

The scores of predicting the associations between two diseases RENAL TUBULAR ACIDOSIS, DISTAL, AUTOSOMAL DOMINANT (OMIM: 179800) as well as OSTEOPETROSIS, AUTOSOMAL RECESSIVE 3; OPTB3 (OMIM: 259730)), and their candidate domains ATP-synt_ab (PF00006), ATP-synt_ab_N (PF02874), Carb_anhydrase (PF00194), HCO3_cotransp (PF00955), Band_3_cyto (PF07565), ATP-synt_ab_C (PF00306), TNF (PF00229), IMPDH (PF00478), CBS (PF00571), Voltage_CLC (PF00654) as well as V_ATPase_I (PF01496), in terms of the Association, MLE (*fp* = 0, *fn* = 0.9), DPEA, Bayesian (*u*_*p*_, *u*_*n*_ = 0, *v*_*p*_, *v*_*n*_ = 1, and *α* = 2, *β* = 2), and PE (*r* = 100 %, and pw threshold ≤ 0.01) approaches, respectively

### Top predictions of domain-disease and gene-disease associations

We list the 50 highest-scoring domain-disease associations predicted using the Bayesian approach, as shown in Table 3. We then map the domains predicted for these diseases to all genes with corresponding proteins containing these domains, and mark the genes that are known as disease genes in the OMIM database using a bold italic style. Among these predictions, 8 are in the above 3004 known domain-disease associations compiled by the Ensembl BioMart tool and we mark them using a bold style in Table 3 (diseases with ranks 6, 7, 9, 19, 20, 25, 42, and 50, respectively). Four of these diseases, CORNEAL DYSTROPHY EPITHELIAL BASEMENT MEMBRANE (EBMD; OMIM: 121820), CORNEAL DYSTROPHY GROENOUW TYPE I (CDGG1; OMIM: 121900), CORNEAL DYSTROPHY LATTICE TYPE I (LCD1; OMIM: 122200) and LUBS X-LINKED MENTAL RETARDATION SYNDROME (MRXSL; OMIM: 300260) have their predicted domains exactly mapped to their only known genes in the OMIM database, while the other 4 diseases have extra predictions of genes that are unknown to be associated with these diseases before. Some of these predictions might be redundant, such as for the disease ALPHA-THALASSEMIA (OMIM: 604131), the gene HBB, which is short for HEMOGLOBIN--BETA LOCUS, is obviously a cause of another type of thalassemia called BETA-THALASSEMIA (OMIM: 613985) but not ALPHA-THALASSEMIA [46]. However, for diseases that without any known genes associated, the predictions might indicate novel genetic findings for the diseases. As listed in the table, all the rows without bold or italic correspond to diseases that have no known OMIM genes at all. But by our methods we can predict for each of them a highest scored domain and a short list of genes, which are highly possible to be the real causal ones since the top ranked diseases are mostly rare diseases and the majority of them are caused by altered functions of single genes [47].

**Table 3:** Novel predictions of domain-disease and gene-disease associations

Rank	Disease	OMIM_d_	Module Index	Domain	Pfam	Gene	OMIM_g_
1	APNEA, OBSTRUCTIVE SLEEP	107650	152	Acetyltransf_1	PF00583	NAA10	300013
						AANAT	600950
2	ARTERIES, ANOMALIES OF	108000	182	Sugar_tr	PF00083	SLC2A1	138140
						SLC2A2	138160
						SLC2A9	606142
						SLC2A10	606145
3	ATRESIA OF EXTERNAL AUDITORY CANAL AND CONDUCTIVE DEAFNESS	108760	133	zf-C2H2_2	PF12756	TSHZ1	614427
4	CELIAC ARTERY STENOSIS FROM COMPRESSION BY MEDIAN ARCUATE LIGAMENT OF DIAPHRAGM	116870	182	Sugar_tr	PF00083	SLC2A1	138140
						SLC2A2	138160
						SLC2A9	606142
						SLC2A10	606145
*5*	*SCHNYDER CORNEAL DYSTROPHY; SCCD*	*121800*	*77*	*Fasciclin*	*PF02469*	*TGFBI*	*601692*
6	CORNEAL DYSTROPHY, EPITHELIAL BASEMENT MEMBRANE; EBMD	121820	77	Fasciclin	PF02469	TGFBI	*601692*
7	CORNEAL DYSTROPHY, GROENOUW TYPE I; CDGG1	121900	77	Fasciclin	PF02469	TGFBI	*601692*
*8*	*CORNEAL DYSTROPHY, MEESMANN; MECD*	*122100*	*77*	*Fasciclin*	*PF02469*	*TGFBI*	*601692*
9	CORNEAL DYSTROPHY, LATTICE TYPE I; LCD1	122200	77	Fasciclin	PF02469	TGFBI	*601692*
10	CORONARY ARTERY DISSECTION, SPONTANEOUS	122455	182	Sugar_tr	PF00083	SLC2A1	138140
						SLC2A2	138160
						SLC2A9	606142
						SLC2A10	606145
11	DEAFNESS, CONDUCTIVE STAPEDIAL, WITH EAR MALFORMATION AND FACIAL PALSY	124490	137	Endothelin	PF00322	EDN1	131240
						EDN3	131242
12	EAR FOLDING	128500	137	Endothelin	PF00322	EDN1	131240
						EDN3	131242
13	PREAURICULAR FISTULAE, CONGENITAL	128700	137	Endothelin	PF00322	EDN1	131240
						EDN3	131242
14	EAR PITS, POSTERIOR HELICAL	128710	137	Endothelin	PF00322	EDN1	131240
						EDN3	131242
*15*	*EAR WITHOUT HELIX*	*128800*	*137*	*Endothelin*	*PF00322*	*EDN1*	*131240*
						*EDN3*	*131242*
16	EXTERNAL AUDITORY CANAL, BILATERAL ATRESIA OF, WITH CONGENITAL VERTICAL TALUS	133705	133	zf-C2H2_2	PF12756	TSHZ1	614427
17	FIBROMUSCULAR DYSPLASIA OF ARTERIES	135580	182	Sugar_tr	PF00083	SLC2A1	138140
						SLC2A2	138160
						SLC2A9	606142
						SLC2A10	606145
18	GLAUCOMA AND SLEEP APNEA	137763	152	Acetyltransf_1	PF00583	NAA10	300013
						AANAT	600950
19	LUBS X-LINKED MENTAL RETARDATION SYNDROME; MRXSL	300260	66	MBD	PF01429	MECP2	*300005*
20	ALPHA-THALASSEMIA	604131	188	Globin	PF00042	HBA1	*141800*
						HBA2	*141850*
						HBB	141900
*21*	*HOLT-ORAM SYNDROME; HOS*	*142900*	*140*	*LMBR1*	*PF04791*	*LMBR1*	*605522*
22	INTERNAL CAROTID ARTERY, SPONTANEOUS DISSECTION OF	147820	182	Sugar_tr	PF00083	SLC2A1	138140
						SLC2A2	138160
						SLC2A9	606142
						SLC2A10	606145
23	LITHIUM TRANSPORT	152420	180	SNF	PF00209	SLC6A3	126455
						SLC6A19	608893
24	MACULAR DYSTROPHY, FENESTRATED SHEEN TYPE	153890	77	Fasciclin	PF02469	TGFBI	601692
25	MULLERIAN APLASIA AND HYPERANDROGENISM	158330	199	wnt	PF00110	WNT5A	164975
						WNT10B	601906
						WNT4	*603490*
						WNT10A	606268
26	OSSIFIED EAR CARTILAGES	165670	137	Endothelin	PF00322	EDN1	131240
						EDN3	131242
27	ENCHONDROMATOSIS, MULTIPLE, OLLIER TYPE	166000	166	Iso_dh	PF00180	IDH2	147650
						IDH1	147700
						IDH3B	604526
*28*	*MACULAR DYSTROPHY, PATTERNED, 1; MDPT1*	*169150*	*77*	*Fasciclin*	*PF02469*	*TGFBI*	*601692*
29	RADIAL RAY HYPOPLASIA WITH CHOANAL ATRESIA	179270	140	LMBR1	PF04791	LMBR1	605522
*30*	*QUESTION MARK EARS, ISOLATED; QME*	*612798*	*137*	*Endothelin*	*PF00322*	*EDN1*	*131240*
						*EDN3*	*131242*
31	THUMB DEFORMITY	188100	140	LMBR1	PF04791	LMBR1	605522
32	THYROID HORMONE PLASMA MEMBRANE TRANSPORT DEFECT	188560	180	SNF	PF00209	SLC6A3	126455
						SLC6A19	608893
33	TRACHEOESOPHAGEAL FISTULA WITH OR WITHOUT ESOPHAGEAL ATRESIA	189960	133, 181	zf-C2H2_2	PF12756	TSHZ1	614427
34	TRIGGER THUMB	190410	140	LMBR1	PF04791	LMBR1	605522
35	TRIPHALANGEAL THUMB WITH DOUBLE PHALANGES	190500	140	LMBR1	PF04791	LMBR1	605522
36	TRIPHALANGEAL THUMB, NONOPPOSABLE	190600	140	LMBR1	PF04791	LMBR1	605522
37	UTERINE ANOMALIES	192000	199	wnt	PF00110	WNT5A	164975
						WNT10B	601906
						WNT4	603490
						WNT10A	606268
38	UTERUS BICORNIS BICOLLIS WITH PARTIAL VAGINAL SEPTUM AND UNILATERAL HEMATOCOLPOS WITH IPSILATERAL RENAL AGENESIS	192050	199	wnt	PF00110	WNT5A	164975
						WNT10B	601906
						WNT4	603490
						WNT10A	606268
39	ACRORENAL-MANDIBULAR SYNDROME	200980	199	wnt	PF00110	WNT5A	164975
						WNT10B	601906
						WNT4	603490
						WNT10A	606268
40	ADDUCTED THUMBS SYNDROME	201550	140	LMBR1	PF04791	LMBR1	605522
41	APNEA, CENTRAL SLEEP	207720	152	Acetyltransf_1	PF00583	NAA10	300013
						AANAT	600950
42	ARTERIAL TORTUOSITY SYNDROME; ATS	208050	182	Sugar_tr	PF00083	SLC2A1	138140
						SLC2A2	138160
						SLC2A9	606142
						SLC2A10	*606145*
43	AURAL ATRESIA, MULTIPLE CONGENITAL ANOMALIES, AND MENTAL RETARDATION	209770	133, 181	zf-C2H2_2	PF12756	TSHZ1	614427
44	BILIARY ATRESIA, EXTRAHEPATIC; EHBA	210500	133, 181	zf-C2H2_2	PF12756	TSHZ1	614427
45	CITRULLINE TRANSPORT DEFECT	215720	180	SNF	PF00209	SLC6A3	126455
						SLC6A19	608893
46	CENTRAL CLOUDY DYSTROPHY OF FRANCOIS; CCDF	217600	77	Fasciclin	PF02469	TGFBI	601692
47	DEAFNESS, CONDUCTIVE, WITH MALFORMED EXTERNAL EAR	221300	137	Endothelin	PF00322	EDN1	131240
						EDN3	131242
*48*	*DICARBOXYLIC AMINOACIDURIA; DCBXA*	*222730*	*180*	*SNF*	*PF00209*	*SLC6A3*	*126455*
						*SLC6A19*	*608893*
49	DUODENAL ATRESIA	223400	181	zf-C2H2_2	PF12756	TSHZ1	614427
50	HARTNUP DISORDER; HND	234500	180	SNF	PF00209	SLC6A3	126455
						SLC6A19	*608893*

“Rank” is the rank of predicted domain-disease associations in terms of Bayesian scores; “Disease” is the name of the disease phenotype, “OMIM_d_” is ID of disease phenotype in the OMIM database; “Module Index” is the index of module including the disease; “Domain” is the name of domain; “Pfam” is the domain ID in the Pfam database; “Gene” is mapped gene from corresponding domain; and “OMIM_g_” is the gene ID in the OMIM database. The bold rows represent known domain-disease associations compiled by the Ensembl BioMart tool. The italic rows represent domain-disease associations that are unknown in our study but have at least one known genes in the OMIM database. The bold italic elements in the “OMIM_g_” column represent that the predicted genes are known as disease genes in the OMIM database

Finally, there are still 7 domain-disease associations that are unknown in our study, but they have at least one known genes in the OMIM database, and we mark them using an italic style (diseases with ranks 5, 8, 15, 21, 28, 30, and 48, respectively). From the prediction result we see that only one of these diseases, QUESTION MARK EARS ISOLATED (QME; OMIM: 612798) successfully recover its known disease genes EDN1 (OMIM: 131240), while the other 6 diseases fail to prioritize their known associated genes at the top. This is one main drawback of the modularization, i.e., it will lose some information of known disease-gene associations when constructing protein-module association. A trade-off is called for to achieve the balance between the exploration of novel disease-gene predictions and the failure of recovering known associations.

### Evidences in genome-wide association studies

Over the past decade, genome-wide association studies (GWAS) have led to the identification of susceptible single nucleotide polymorphisms (SNPs) conferring risk for common human diseases [48]. The NHGRI-EBI catalog of published genome-wide association studies [3] is a quality controlled, manually curated, literature-derived collection of all published genome-wide association studies assaying at least 100,000 SNPs and all SNP-trait associations with *p*-values < 1.0 × 10^− 5^ [49]. With this resource, it is of interest to test the consistency of our inferred domain-disease associations with the GWAS results.

We choose the inference results obtained using the Bayesian approach for further analysis since it performs the best in the above validation experiments. Then for a given disease of interest, from the GWAS catalog we are able to collect a list of reported susceptible SNPs, and check how many of these SNPs appear within 5 Mbp around the domains that are ranked in top 10 in our inference results. We next select Crohn’s disease (OMIM: 266600) and Type 2 diabetes (OMIM: 125853) as two typical examples of common polygenetic disorders with multiple genes contributing to the diseases, therefore GWAS studies provide a collection of risk factors for these two disorders.

#### Crohn’s disease

Crohn’s disease is a type of chronic inflammatory bowel disease (IBD) of the gastrointestinal tract [50] affecting 26–200 per 100,000 in European populations [51], and is thought to be caused by a combination of environmental, immune and bacterial factors in genetically susceptible individuals [52]. Recently, genome-wide association studies (GWAS) have made remarkable progress in Crohn’s disease identifying at least 140 genome-wide significant loci [53]. In our study, we compile from GWAS catalog 203 reported susceptible SNPs, and 54 of them are found to be within 5 Mbp regions of all domains that are ranked at the top 10. Then for each of the top 10 domains, we check regions of their corresponding genes, to see if any GWAS reported SNPs are inside, upstream, or downstream within 5Mbp of every gene region, and list the results in Additional file 1: Table S4.

From the table we observe 18 times that a susceptible SNP locates inside a domain with 10 times in ATG16 (PF08614), 4 times in NACHT (PF05729), and 4 times in CARD (PF00619). We also observe 24 times that a susceptible SNP locates within 1 Mbp upstream or downstream of a domain, 52 times that a susceptible SNP locates within 1 to 5 Mbp region of a domain, and only 33 times that a susceptible SNP locates beyond 5 Mbp region of a domain. From these results we conjecture that domains ranked among the top 10 indeed tends to be closer to, even include, known susceptible SNPs for this disease.

#### Type 2 diabetes

Type 2 diabetes, which is also called noninsulin-dependent diabetes mellitus (NIDDM) or adult-onset diabetes, is a metabolic disorder that is characterized by high blood sugar in the context of insulin resistance and relative lack of insulin [54]. It results from interaction between genetic and environmental risk factors [55–57]. To uncover the genetic basis of the disease, at least 88 SNPs have been reported being associated with increased risk for Type 2 diabetes [58]. In our study, we compile from GWAS catalog 221 reported susceptible SNPs, and 35 of them are found to be within 5 Mbp regions of 8 domains that are ranked at the top 10 (except domain Peptidase_M10 (PF00413) and LTD (PF00932). Similarly, for each of the top 10 domains, we check regions of their corresponding genes, to see if any GWAS reported SNPs are inside, upstream, or downstream within 5Mbp of every gene region, and list the results in Additional file 1: Table S5.

From the table we observe 3 times that a susceptible SNP locates inside a domain with once in PH (PF00169) and twice in IRK (PF01007). We also observe 19 times that a susceptible SNP locates within [13] 1 Mbp upstream or downstream of a domain, 32 times that a susceptible SNP locates within 1 to 5 Mbp region of a domain, and also 32 times that a susceptible SNP locates beyond 5 Mbp region of a domain. From these results we conjecture that domains ranked among the top 10 indeed tends to be closer to, and even include, known susceptible SNPs for this disease.

## Conclusions and discussion

In this paper, we implemented a comparative study of inferring protein domains that are associated with human inherited diseases, by means of five approaches that are previously used to predict domain-domain interactions from protein-protein interactions. On the basis of several network data sources, we first constructed disease modules from the disease phenotype similarity network, and then proposed a framework to make the inference through known relations between diseases and modules, domains and proteins, as well as proteins and disease modules. We further demonstrate the effectiveness and robustness of these approaches, through a series of large-scale validation experiments, and discussed about the benefits brought by modularization, while comparing the performances of the five approaches in terms of three evaluation criteria (AUC score, Accuracy, and Mean rank ratio). We finally illustrate the consistency between our inference results and the evidences from genome-wide association studies for two common diseases: Crohn’s disease and Type 2 diabetes.

Our main contribution therefore lies in the following parts: (1) effectively utilization of protein-domain, gene-disease and phenotype similarity sources to make large-scale inference of domain-disease associations and even novel gene-disease associations; (2) comparisons of the predictive powers of five well-known approaches in solving the domain-disease association inference problem; (3) comprehensive analysis of the robustness of the approaches to changing parameters; (4) illustration of the effects of disease modularization in predicting novel domain-disease and gene-disease associations; (5) analysis of top 50 predictions of domain-disease and corresponding gene-disease associations; (6) demonstration of the consistency of our inference results with the genome-wide association studies, providing genetic support to our domain-disease association inference results.

Notwithstanding so, our work can be further explored from the following aspects. First, the success of all the five approaches is mainly owing to the known gene-disease, protein-domain relationships and the construction of disease modules based on phenotype similarities. Therefore, the abundances and qualities of these relationships are of great importance to the inference results. However, current studies concerning the structures and functions protein domains are still too few, making the domain-centric approach instead of the gene-centric ones challenging [13]. To improve this situation one may resort to more network containing the domain information as an alternative, such as the domain-domain interactions from the DOMINE database [59]. As for the construction of disease module, although there are already several methods for calculating similarities between disease phenotypes, how to categorize the diseases remains a challenge. In our work we just simply construct the phenotype similarity network according to a threshold, through which the numerical information of similarities among diseases is not sufficiently utilized. The threshold itself needs to be pre-determined as well, and in this paper we select its value mainly for the consideration of computational feasibility. However, different values of the threshold would change the number of diseases, proteins, and domains involved, and different clustering approaches and their potential parameters used may also influence significantly. For clustering diseases into biological meaningful modules, more knowledge about disease categorization is needed.

Second, although we successfully developed the disease modules to circumvent the limitations of the insufficient protein-disease associations, the modularization does not differentiate the diseases in the same modules, and known protein-disease information is reduced to protein-module information instead. As a result, for the diseases that already have known genes associated, these disease genes are not guaranteed to be scored highest for the diseases. Therefore, to overcome this problem, we need to assign different weights to diseases in the same module during the disease modularization process.

Third, as shown in Table 4, we list the strengths and weaknesses for all the five approaches referred in this paper. Although the Bayesian approach performs the best in our comparative study, it also suffers from some mathematical and computational issues that need to be improved upon. Although the Adaptive Rejection Sampling method in the Bayesian approach only needs that the posterior distribution to be log-concave, we still need to determine the prior distributions for the parameters involved. As is known, the specification of prior is intrinsically complicated and subjective. The main consideration is that the posterior mean and variance should not depend on the units in which the disease similarities are measured and should also be invariant to the shift of the response variable. Therefore, one can consider the use of Jeffrey’s prior instead of the conjugate prior. Also, the computational time of the Bayesian approach is much longer than the other four approaches, and as the scale of networks increase, the iteration processes becomes unbearable. More computational efficient approaches to estimate the posterior probability distributions are needed.

**Table 4:** Strengths and weaknesses of the approaches

	Strength	Weakness
Association	• Fast in implementation • No need to pre-determine parameters • Results can be easily validated by hand calculation	• Unsatisfactory in predictive power • Does not consider the structures all relevant protein-module associations as a whole • Do not have control for possible over-prediction of associations between frequently occurring domain-disease pairs
MLE	• Good in predictive power • Fast in implementation • Take into account the structures all relevant protein-module associations as a whole	• Need to pre-determine parameters • Do not have control for possible over-prediction of associations between frequently occurring domain-disease pairs
DPEA	• Satisfactory in predictive power • No need to pre-determine parameters • Have control for possible over-prediction of associations between frequently occurring domain-disease pairs	• Slow in implementation when the number of candidate domain-disease associations is large
Bayesian	• Excellent in predictive power • Take into account the structures all relevant protein-module associations as a whole	• Slow in implementation when the number of candidate domain-disease associations is large • Do not have control for possible over-prediction of associations between frequently occurring domain-disease pairs • Failure when the log-concave conditions of parameters are not satisfied
PE	• Satisfactory in predictive power • Only one pre-determined parameter • Have control for possible over-prediction of associations between frequently occurring domain-disease pairs	• Slow in implementation when the number of candidate domain-disease associations is large

Finally, our study is mainly motivated by the hypothesis that protein domains can be viewed as functional units of proteins, and are considered important in disease development. However, in addition to domains, the linker sequences between domains could also be essential for the biological functions of the proteins, which has been demonstrated by a recent study [60]. Some linker sequences can be viewed as flexible “domains”, by connecting various domains in a single protein without interfering with the function of each domain [61], and their relationships between linker sequences and diseases is a topic for a future research.

## Additional files

Additional file 1: Table S1. Number of domains, diseases, proteins and modules collected based on different thresholds used in the disease module identification. **Table S2**: The performances as measured by AUC, accuracy and mean rank ratio of the MLE approach under different combinations of the false positive rate *fp* and the false negative rate *fn*. **Table S3**: The performances as measured by AUC, accuracy and mean rank ratio of the PE approach under different reliability rate and pw-score threshold. **Table S4**: GWAS evidence between domains and Crohn’s disease. **Table S5**: GWAS evidence between domains and Type 2 Diabetes. (DOCX 47 kb) Additional file 2:
**Supplemental methods.** This file contains a) symbols used in the equations and formulations of the methods, b) details of implementing the EM algorithm in the maximum likelihood estimation (MLE) approach, and c) details of deducing the score function in the domain-disease pair exclusion analysis (DPEA) approach. (DOCX 197 kb)
