# Supplementary material for: Inference of domain-disease associations from domain-protein, protein-disease and disease-disease relationships
Source: BMC Syst Biol. 2016 Jan 11;10(Suppl 1):4. doi: 10.1186/s12918-015-0247-y (PMC4895779; doi:10.1186/s12918-015-0247-y)
Supplement: Additional file 1: Table S1. — Number of domains, diseases, proteins and modules collected based on different thresholds used in the disease module identification. Table S2: The performances as measured by AUC, accuracy and mean rank ratio of the MLE approach under different combinations of the false positive rate fp and the false negative rate fn. Table S3: The performances as measured by AUC, accuracy and mean rank ratio of the PE approach under different reliability rate and pw-score threshold. Table S4: GWAS evidence between domains and Crohn’s disease. Table S5: GWAS evidence between domains and Type 2 Diabetes. (DOCX 47 kb) [file 12918_2015_247_MOESM1_ESM.docx]

# Additional file 2: Supplemental tables

**Table S1 – Number of domains, diseases, proteins and modules collected based on different threshold used in the disease module identification.** The threshold 0.5 is used in this paper.

| Threshold | # Domains | # Diseases | # Proteins | # Modules |
| --- | --- | --- | --- | --- |
| 0.1 | 301 | 428 | 273 | 2 |
| 0.2 | 767 | 2054 | 706 | 6 |
| 0.3 | 1075 | 3757 | 1156 | 56 |
| 0.4 | 1145 | 4090 | 1298 | 174 |
| **0.5** | **1106** | **3430** | **1238** | **255** |
| 0.6 | 880 | 2304 | 936 | 228 |
| 0.7 | 589 | 1209 | 557 | 147 |
| 0.8 | 205 | 311 | 152 | 45 |
| 0.9 | 41 | 29 | 23 | 7 |

**Table S2 – The performances as measured by AUC, accuracy and mean rank ratio of the MLE approach under different combinations of the false positive rate *fp* and the false negative rate *fn*.** The shading areas show that when both *fp* and *fn* are relatively large, one can observe significant lower AUCs and accuracies, as well as higher mean rank ratios.

| AUC | | *fn* | | | | | | | | | |
| --- | --- | --- | --- | --- | --- | --- | --- | --- | --- | --- | --- |
|  |  | 0 | 0.1 | 0.2 | 0.3 | 0.4 | 0.5 | 0.6 | 0.7 | 0.8 | 0.9 |
| *fp* | 0 | 0.8322 | 0.8313 | 0.8313 | 0.8308 | 0.8308 | 0.8308 | 0.8316 | 0.8366 | 0.8392 | 0.8407 |
|  | 0.1 | 0.8321 | 0.8312 | 0.8312 | 0.8312 | 0.8312 | 0.8308 | 0.8316 | 0.8321 | 0.8369 | 0.8374 |
|  | 0.2 | 0.8321 | 0.8312 | 0.8312 | 0.8312 | 0.8312 | 0.8308 | 0.8315 | 0.8321 | 0.8368 | 0.4919 |
|  | 0.3 | 0.8316 | 0.8308 | 0.8308 | 0.8308 | 0.8308 | 0.8308 | 0.8294 | 0.8167 | 0.5205 | 0.4927 |
|  | 0.4 | 0.8282 | 0.8277 | 0.8277 | 0.8277 | 0.8277 | 0.8233 | 0.8158 | 0.6020 | 0.5288 | 0.4935 |
|  | 0.5 | 0.8208 | 0.8175 | 0.8175 | 0.8175 | 0.8175 | 0.8155 | 0.6712 | 0.6156 | 0.5341 | 0.4963 |
|  | 0.6 | 0.8198 | 0.8141 | 0.8141 | 0.8043 | 0.8070 | 0.7167 | 0.6809 | 0.6249 | 0.5441 | 0.5009 |
|  | 0.7 | 0.8198 | 0.8135 | 0.8027 | 0.7963 | 0.7923 | 0.7174 | 0.6803 | 0.6260 | 0.5504 | 0.5095 |
|  | 0.8 | 0.8144 | 0.8018 | 0.7954 | 0.7923 | 0.7911 | 0.7180 | 0.6803 | 0.6259 | 0.5684 | 0.5103 |
|  | 0.9 | 0.7978 | 0.7950 | 0.7944 | 0.7937 | 0.7923 | 0.7147 | 0.6803 | 0.6247 | 0.5759 | 0.5172 |
| Accuracy | | *fn* | | | | | | | | | |
|  |  | 0 | 0.1 | 0.2 | 0.3 | 0.4 | 0.5 | 0.6 | 0.7 | 0.8 | 0.9 |
| *fp* | 0 | 0.6968 | 0.6935 | 0.6935 | 0.6927 | 0.6927 | 0.6927 | 0.6960 | 0.6985 | 0.7041 | 0.7074 |
|  | 0.1 | 0.6968 | 0.6935 | 0.6935 | 0.6935 | 0.6935 | 0.6927 | 0.6960 | 0.6968 | 0.7009 | 0.7016 |
|  | 0.2 | 0.6968 | 0.6935 | 0.6935 | 0.6935 | 0.6935 | 0.6927 | 0.6960 | 0.6968 | 0.7004 | 0.2669 |
|  | 0.3 | 0.6960 | 0.6927 | 0.6927 | 0.6927 | 0.6927 | 0.6927 | 0.6918 | 0.6448 | 0.3698 | 0.2967 |
|  | 0.4 | 0.6885 | 0.6882 | 0.6882 | 0.6882 | 0.6882 | 0.6874 | 0.6436 | 0.4565 | 0.4034 | 0.3040 |
|  | 0.5 | 0.6870 | 0.6514 | 0.6514 | 0.6514 | 0.6514 | 0.6436 | 0.5738 | 0.4658 | 0.4115 | 0.3090 |
|  | 0.6 | 0.6870 | 0.6412 | 0.6412 | 0.6195 | 0.5963 | 0.5910 | 0.5847 | 0.4749 | 0.4103 | 0.3154 |
|  | 0.7 | 0.6539 | 0.6412 | 0.6220 | 0.6097 | 0.6056 | 0.5913 | 0.5921 | 0.4749 | 0.4131 | 0.3252 |
|  | 0.8 | 0.6417 | 0.6208 | 0.6085 | 0.6056 | 0.6052 | 0.5921 | 0.5921 | 0.4749 | 0.4140 | 0.3277 |
|  | 0.9 | 0.6119 | 0.6080 | 0.6069 | 0.6061 | 0.6056 | 0.5880 | 0.5921 | 0.4741 | 0.4198 | 0.3310 |
| Mean  rank ratio | | *fn* | | | | | | | | | |
|  |  | 0 | 0.1 | 0.2 | 0.3 | 0.4 | 0.5 | 0.6 | 0.7 | 0.8 | 0.9 |
| *fp* | 0 | 0.2079 | 0.2128 | 0.2128 | 0.2142 | 0.2142 | 0.2142 | 0.2094 | 0.2055 | 0.1967 | 0.1914 |
|  | 0.1 | 0.2079 | 0.2128 | 0.2128 | 0.2128 | 0.2128 | 0.2142 | 0.2094 | 0.2079 | 0.2016 | 0.2006 |
|  | 0.2 | 0.2079 | 0.2128 | 0.2128 | 0.2128 | 0.2128 | 0.2142 | 0.2103 | 0.2084 | 0.2021 | 0.5778 |
|  | 0.3 | 0.2094 | 0.2137 | 0.2142 | 0.2142 | 0.2142 | 0.2147 | 0.2157 | 0.2546 | 0.4170 | 0.5317 |
|  | 0.4 | 0.2205 | 0.2215 | 0.2215 | 0.2215 | 0.2215 | 0.2225 | 0.2551 | 0.3825 | 0.4650 | 0.5190 |
|  | 0.5 | 0.2235 | 0.2330 | 0.2388 | 0.2388 | 0.2388 | 0.2551 | 0.2634 | 0.3674 | 0.4524 | 0.5117 |
|  | 0.6 | 0.2235 | 0.2580 | 0.2580 | 0.2580 | 0.2643 | 0.2867 | 0.2860 | 0.3538 | 0.4534 | 0.5049 |
|  | 0.7 | 0.2345 | 0.2580 | 0.2781 | 0.2771 | 0.2834 | 0.2962 | 0.2948 | 0.3538 | 0.4499 | 0.4865 |
|  | 0.8 | 0.2575 | 0.2701 | 0.2790 | 0.2834 | 0.2834 | 0.2948 | 0.2948 | 0.3538 | 0.4480 | 0.4821 |
|  | 0.9 | 0.2737 | 0.2800 | 0.2815 | 0.2819 | 0.2834 | 0.2911 | 0.2948 | 0.3548 | 0.4398 | 0.4777 |

**Table S3 – The performances as measured by AUC, accuracy and mean rank ratio of the PE approach under different reliability rate and pw-score threshold.**

| pw-score threshold | Reliability rate | AUC | Accuracy | Mean rank ratio |
| --- | --- | --- | --- | --- |
| 0.01 | 100% | 0.8262 | 0.6525 | 0.2282 |
|  | 90% | 0.8229 | 0.6485 | 0.2397 |
|  | 80% | 0.8195 | 0.6392 | 0.2563 |
|  | 70% | 0.8049 | 0.6242 | 0.2666 |
|  | 60% | 0.7852 | 0.6018 | 0.2907 |
|  | 50% | 0.7635 | 0.5793 | 0.2929 |
| 0.05 | 100% | 0.8238 | 0.6509 | 0.2314 |
|  | 90% | 0.8222 | 0.6478 | 0.2408 |
|  | 80% | 0.8171 | 0.6377 | 0.2574 |
|  | 70% | 0.8077 | 0.6194 | 0.2680 |
|  | 60% | 0.7840 | 0.5982 | 0.2910 |
|  | 50% | 0.7648 | 0.5763 | 0.2933 |
| 0.10 | 100% | 0.8235 | 0.6499 | 0.2365 |
|  | 90% | 0.8212 | 0.6462 | 0.2485 |
|  | 80% | 0.8169 | 0.6371 | 0.2619 |
|  | 70% | 0.8004 | 0.6168 | 0.2721 |
|  | 60% | 0.7856 | 0.5976 | 0.2956 |
|  | 50% | 0.7602 | 0.5683 | 0.2997 |

**Table S4 – GWAS evidence between domains and Crohn’s disease.** “Rank” denotes the rank of the corresponding domain in the inference of domain-disease associations. “Domain” denotes the Pfam ID of the domain. “Chr” denotes the chromosome at which the domain locates. “Region” denotes predicted regions that may include susceptible SNPs (Mb means 10E6 base pairs). “SNP” denotes reported susceptible SNP from the literature or databases. “Position” denotes position of the susceptible SNP. “Distance” denotes the distance from the susceptible SNP to the predicted domain region.

| Rank | Domain | Chr | Region (Mb) | SNP | Position (Mb) | Distance |
| --- | --- | --- | --- | --- | --- | --- |
| 1 | PF07714 | 1 | 16.124337 – 16.156087 | rs7667 | 19.39233 | 3.236243 Mb down |
|  |  |  | 22.710839 – 22.921500 | rs7667 | 19.39233 | 3.318509 Mb up |
|  |  |  |  | rs7551188 | 24.946709 | 2.025209 Mb down |
|  |  | 2 | 29.192774 – 29.921566 | rs1728918 | 27.412596 | 1.780178 Mb up |
|  |  |  |  | rs13428812 | 25.269598 | 3.923176 Mb up |
|  |  |  |  | rs780093 | 27.519736 | 1.673038 Mb up |
|  |  |  | 111.898479 – 112.029561 | -- | -- | > 5Mb |
|  |  | 4 | 1.793307 – 1.808872 | -- | -- | > 5Mb |
|  |  | 5 | 180.601506 – 180.649624 | -- | -- | > 5Mb |
|  |  | 7 | 55.019021 – 55.256620 | rs1456893 | 50.230076 | 4.788945 Mb up |
|  |  |  |  | rs1456896 | 50.264865 | 4.754156 Mb up |
|  |  |  | 116.672390 – 116.798386 | rs1869839 | 114.717488 | 1.954902 Mb up |
|  |  |  | 140.719327 – 140.924764 | -- | -- | > 5Mb |
|  |  | 8 | 11.494001 – 11.564604 | -- | -- | > 5Mb |
|  |  |  | 38.411138 – 38.468834 | -- | -- | > 5Mb |
|  |  | 9 | 110.668771 – 110.801579 | rs6478106 | 114.783386 | 3.981807 Mb down |
|  |  |  |  | rs3810936 | 114.790605 | 3.989026 Mb down |
|  |  |  |  | rs4263839 | 114.80416 | 4.002581 Mb down |
|  |  | 10 | 43.077027 – 43.130351 | -- | -- | > 5Mb |
|  |  |  | 121.478334 – 121.598458 | -- | -- | > 5Mb |
|  |  | 12 | 56.079857 – 56.103505 | -- | -- | > 5Mb |
|  |  | 17 | 39.687914 – 39.730426 | rs2872507 | 39.88451 | 0.154084 Mb down |
|  |  |  |  | rs9891119 | 42.355962 | 2.625536 Mb down |
|  |  |  |  | rs744166 | 42.362183 | 2.631757 Mb down |
|  |  |  |  | rs11871801 | 42.418754 | 2.688328 Mb down |
|  |  | 19 | 17.824780 – 17.848071 | -- | -- | > 5Mb |
|  |  | X | 101.349447 – 101.390796 | -- | -- | > 5Mb |
| 2 | PF01391 | 1 | 22.652762 – 22.661538 | rs7667 | 19.39233 | 3.260432 Mb up |
|  |  |  |  | rs7551188 | 24.946709 | 2.285171 Mb down |
|  |  |  | 27.369112 – 27.374824 | rs7551188 | 24.946709 | 2.422403 Mb up |
|  |  |  | 36.095236 – 36.125220 | -- | -- | > 5Mb |
|  |  |  | 102.876467 – 103.108496 | -- | -- | > 5Mb |
|  |  | 2 | 237.324003 – 237.414375 | rs10210302 | 233.250193 | 4.07381 Mb up |
|  |  |  |  | rs12994997 | 233.264857 | 4.059146 Mb up |
|  |  |  |  | rs3828309 | 233.271764 | 4.052239 Mb up |
|  |  |  |  | rs2241880 | 233.274722 | 4.049281 Mb up |
|  |  |  |  | rs3792109 | 233.275771 | 4.048232 Mb up |
|  |  | 3 | 48.564073 – 48.595267 | rs9858542 | 49.66455 | 1.069283 Mb down |
|  |  |  |  | rs3197999 | 49.684099 | 1.088832 Mb down |
|  |  |  | 186.842690 – 186.858463 | -- | -- | > 5Mb |
|  |  | 6 | 33.162681 – 33.192499 | rs9258260 | 29.755384 | 3.407297 Mb up |
|  |  |  |  | rs3094188 | 31.174468 | 1.988213 Mb up |
|  |  |  |  | rs9264942 | 31.306603 | 1.856078 Mb up |
|  |  |  |  | rs1799964 | 31.574531 | 1.58815 Mb up |
|  |  |  |  | rs9348876 | 31.607499 | 1.555182 Mb up |
|  |  |  |  | rs9267911 | 32.237333 | 0.925348 Mb up |
|  |  |  |  | rs10947261 | 32.405455 | 0.757226 Mb up |
|  |  |  |  | rs9271366 | 32.619077 | 0.543604 Mb up |
|  |  |  |  | rs9469220 | 32.690533 | 0.472148 Mb up |
|  |  |  |  | rs7765379 | 32.713151 | 0.44953 Mb up |
|  |  |  |  | rs751728 | 33.796256 | 0.603757 Mb down |
|  |  | 7 | 94.394561 – 94.431232 | -- | -- | > 5Mb |
|  |  | 8 | 16.107878 – 16.567490 | -- | -- | > 5Mb |
|  |  | 10 | 79.555852 – 79.560397 | rs1250544 | 79.273128 | 0.282724 Mb up |
|  |  |  |  | rs1250550 | 79.30056 | 0.255292 Mb up |
|  |  |  | 79.610939 – 79.615455 | rs1250544 | 79.273128 | 0.337811 Mb up |
|  |  |  |  | rs1250550 | 79.30056 | 0.310379 Mb up |
|  |  | 17 | 50.183289 – 50.201632 | -- | -- | > 5Mb |
|  |  | 21 | 46.098097 – 46.132849 | rs2838519 | 44.19514 | 1.902957 Mb up |
|  |  |  |  | rs762421 | 44.195678 | 1.902419 Mb up |
|  |  | X | 69.616067 – 70.039469 | -- | -- | > 5Mb |
|  |  |  | 108.155607 – 108.439497 | -- | -- | > 5Mb |
| 3 | PF00489 | 7 | 22.725884 – 22.732002 | rs10486483 | 26.852821 | 4.120819 Mb down |
| 4 | PF08614 | 2 | 233.210051 – 233.295674 | rs12994997 | 233.264857 | inside |
|  |  |  |  | rs6716753 | 230.232414 | inside |
|  |  |  |  | rs3792109 | 233.275771 | inside |
|  |  |  |  | rs2241880 | 233.274722 | inside |
|  |  |  |  | rs3792109 | 233.275771 | inside |
|  |  |  |  | rs7423615 | 230.252159 | inside |
|  |  |  |  | rs2241880 | 233.274722 | inside |
|  |  |  |  | rs3828309 | 233.271764 | inside |
|  |  |  |  | rs10210302 | 233.250193 | inside |
|  |  |  |  | rs2241880 | 233.274722 | inside |
| 5 | PF05729 | 16 | 50.693603 – 50.733077 | rs2066847 | 50.729867 | inside |
|  |  |  |  | rs2076756 | 50.72297 | inside |
|  |  |  |  | rs5743289 | 50.722863 | inside |
|  |  |  |  | rs17221417 | 50.705671 | inside |
| 6 | PF07686 | 1 | 161.304735 – 161.309972 | rs4656940 | 160.860478 | 0.444257 Mb up |
|  |  |  |  | rs2274910 | 160.882256 | 0.422479 Mb up |
|  |  | 2 | 86.784610 – 86.808396 | -- | -- | > 5Mb |
|  |  |  | 203.867786 – 203.873960 | -- | -- | > 5Mb |
|  |  |  | 241.849881 – 241.858908 | -- | -- | > 5Mb |
|  |  | 6 | 29.656981 – 29.672372 | rs9258260 | 29.755384 | 0.083012 Mb down |
|  |  |  |  | rs3094188 | 31.174468 | 1.502096 Mb down |
|  |  |  |  | rs9264942 | 31.306603 | 1.634231 Mb down |
|  |  |  |  | rs1799964 | 31.574531 | 1.902159 Mb down |
|  |  |  |  | rs9348876 | 31.607499 | 1.935127 Mb down |
|  |  |  |  | rs9267911 | 32.237333 | 2.564961 Mb down |
|  |  |  |  | rs10947261 | 32.405455 | 2.733083 Mb down |
|  |  |  |  | rs9271366 | 32.619077 | 2.946705 Mb down |
|  |  |  |  | rs9469220 | 32.690533 | 3.018161 Mb down |
|  |  |  |  | rs7765379 | 32.713151 | 3.040779 Mb down |
|  |  |  |  | rs751728 | 33.796256 | 4.123884 Mb down |
|  |  | 11 | 118.133377 – 118.152888 | -- | -- | > 5Mb |
|  |  |  | 118.161951 – 118.176673 | -- | -- | > 5Mb |
|  |  |  | 123.629187 – 123.655244 | -- | -- | > 5Mb |
| 7 | PF00017 | 2 | 97.713560 – 97.739862 | rs2058660 | 102.437989 | 4.698127 Mb down |
|  |  |  | 191.029576 – 191.151596 | -- | -- | > 5Mb |
|  |  | 5 | 68.215720 – 68.301821 | rs7702331 | 73.255307 | 4.953486 Mb down |
|  |  |  | 87.267888 – 87.391931 | -- | -- | > 5Mb |
|  |  | 8 | 11.494001 – 11.564604 | -- | -- | > 5Mb |
|  |  | 12 | 112.418351 – 112.509913 | -- | -- | > 5Mb |
|  |  | X | 101.349447 – 101.390796 | -- | -- | > 5Mb |
| 8 | PF01108 | 21 | 33.266358 – 33.297234 | rs9258260 | 29.755384 | 3.510974 Mb up |
|  |  |  |  | rs3094188 | 31.174468 | 2.09189 Mb up |
|  |  |  |  | rs9264942 | 31.306603 | 1.959755 Mb up |
|  |  |  |  | rs1799964 | 31.574531 | 1.691827 Mb up |
|  |  |  |  | rs9348876 | 31.607499 | 1.658859 Mb up |
|  |  |  |  | rs9267911 | 32.237333 | 1.029025 Mb up |
|  |  |  |  | rs10947261 | 32.405455 | 0.860903 Mb up |
|  |  |  |  | rs9271366 | 32.619077 | 0.647281 Mb up |
|  |  |  |  | rs9469220 | 32.690533 | 0.575825 Mb up |
|  |  |  |  | rs7765379 | 32.713151 | 0.553207 Mb up |
|  |  |  |  | rs751728 | 33.796256 | 0.499022 Mb down |
| 9 | PF00605 | 1 | 209.785623 – 209.806175 | rs3024505 | 206.766559 | 3.019064 Mb up |
|  |  | 5 | 132.481609 – 132.490798 | rs3091338 | 132.067045 | 0.414564 Mb up |
|  |  |  |  | rs6596075 | 132.406536 | 0.075073 Mb up |
|  |  |  |  | rs2188962 | 132.435113 | 0.046496 Mb up |
|  |  |  |  | rs12521868 | 132.448701 | 0.032908 Mb up |
|  |  | 7 | 128.937612 – 128.950035 | -- | -- | > 5Mb |
| 10 | PF00619 | 7 | 2.906141 – 3.043945 | -- | -- | > 5Mb |
|  |  | 12 | 93.677375 – 93.894840 | -- | -- | > 5Mb |
|  |  | 16 | 50.693603 – 50.733077 | rs2066847 | 50.729867 | inside |
|  |  |  |  | rs2076756 | 50.72297 | inside |
|  |  |  |  | rs5743289 | 50.722863 | inside |
|  |  |  |  | rs17221417 | 50.705671 | inside |
|  |  |  | 67.170154 – 67.175735 | rs11574514 | 67.937477 | 0.761742 Mb down |

**Table S5 – GWAS evidence between domains and Type 2 Diabetes.** “Rank” denotes the rank of the corresponding domain in the inference of domain-disease associations. “Domain” denotes the Pfam ID of the domain. “Chr” denotes the chromosome at which the domain locates. “Region” denotes predicted regions that may include susceptible SNPs (Mb means 10E6 base pairs). “SNP” denotes reported susceptible SNP from the literature or databases. “Position” denotes position of the susceptible SNP. “Distance” denotes the distance from the susceptible SNP to the predicted domain region.

| Rank | Domain | Chr | Region (Mb) | SNP | Position (Mb) | Distance |
| --- | --- | --- | --- | --- | --- | --- |
| 1 | PF07679 | 1 | 77.888513 – 77.943895 | -- | -- | > 5Mb |
|  |  | 2 | 111.898479 – 112.029561 | -- | -- | > 5Mb |
|  |  |  | 178.525989 – 178.830802 | -- | -- | > 5Mb |
|  |  | 3 | 75.906695 – 77.649964 | -- | -- | > 5Mb |
|  |  | 4 | 1.793307 – 1.808872 | rs2290402 | 0.94773 | 0.845577 Mb up |
|  |  |  |  | rs7656416 | 1.260747 | 0.53256 Mb up |
|  |  |  |  | rs6815464 | 1.316113 | 0.477194 Mb up |
|  |  |  |  | rs4689388 | 6.268329 | 4.459457 Mb down |
|  |  |  |  | rs4458523 | 6.288259 | 4.479387 Mb down |
|  |  |  |  | rs1801214 | 6.301295 | 4.492423 Mb down |
|  |  | 5 | 137.867791 – 137.887851 | rs319598 | 134.904545 | 2.963246 Mb up |
|  |  |  | 180.601506 – 180.649624 | -- | -- | > 5Mb |
|  |  | 8 | 38.411138 – 38.468834 | rs516946 | 41.66173 | 3.192896 Mb down |
|  |  |  |  | rs515071 | 41.661944 | 3.19311 Mb down |
|  |  | 9 | 110.668771 – 110.801579 | rs1327796 | 109.764009 | 0.904762 Mb up |
|  |  |  |  | rs10980508 | 110.657479 | 0.011292 Mb up |
|  |  | 10 | 68.106117 – 68.212017 | rs1802295 | 69.171718 | 0.959701 Mb down |
|  |  |  |  | rs2812533 | 69.692529 | 1.480512 Mb down |
|  |  |  | 121.478334 – 121.598458 | rs10886471 | 119.389891 | 2.088443 Mb up |
|  |  |  |  | rs10510110 | 122.432914 | 0.834456 Mb down |
|  |  | 11 | 47.331397 – 47.352702 | -- | -- | > 5Mb |
|  |  |  | 124.865386 – 124.881470 | rs7107217 | 129.603795 | 4.722325 Mb down |
|  |  |  | 132.414977 – 133.532519 | rs7107217 | 129.603795 | 2.811182 Mb up |
|  |  | 12 | 101.568353 – 101.686018 | -- | -- | > 5Mb |
|  |  | 13 | 123.610049 – 123.884331 | -- | -- | > 5Mb |
| 2 | PF07714 | 1 | 16.124337 – 16.156087 | -- | -- | > 5Mb |
|  |  |  | 22.710839 – 22.921500 | -- | -- | > 5Mb |
|  |  | 2 | 29.192774 – 29.921566 | rs12613372 | 30.845153 | 0.923587 Mb down |
|  |  |  | 111.898479 – 112.029561 | -- | -- | > 5Mb |
|  |  | 4 | 1.793307 – 1.808872 | rs2290402 | 0.94773 | 0.845577 Mb up |
|  |  |  |  | rs7656416 | 1.260747 | 0.53256 Mb up |
|  |  |  |  | rs6815464 | 1.316113 | 0.477194 Mb up |
|  |  |  |  | rs4689388 | 6.268329 | 4.459457 Mb down |
|  |  |  |  | rs4458523 | 6.288259 | 4.479387 Mb down |
|  |  |  |  | rs1801214 | 6.301295 | 4.492423 Mb down |
|  |  | 5 | 180.601506 – 180.649624 | -- | -- | > 5Mb |
|  |  | 7 | 55.019021 – 55.256620 | -- | -- | > 5Mb |
|  |  |  | 116.672390 – 116.798386 | -- | -- | > 5Mb |
|  |  |  | 140.719327 – 140.924764 | -- | -- | > 5Mb |
|  |  | 8 | 11.494001 – 11.564604 | -- | -- | > 5Mb |
|  |  |  | 38.411138 – 38.468834 | rs516946 | 41.66173 | 3.192896 Mb down |
|  |  |  |  | rs515071 | 41.661944 | 3.19311 Mb down |
|  |  | 9 | 110.668771 – 110.801579 | rs1327796 | 109.764009 | 0.904762 Mb up |
|  |  |  |  | rs10980508 | 110.657479 | 0.011292 Mb up |
|  |  | 10 | 43.077027 – 43.130351 | -- | -- | > 5Mb |
|  |  |  | 121.478334 – 121.598458 | rs10886471 | 119.389891 | 2.088443 Mb up |
|  |  |  |  | rs10510110 | 122.432914 | 0.834456 Mb down |
|  |  | 12 | 56.079857 – 56.103505 | rs1153188 | 54.705212 | 1.374645 Mb up |
|  |  | 17 | 39.687914 – 39.730426 | rs4430796 | 37.738049 | 1.949865 Mb up |
|  |  | 19 | 17.824780 – 17.848071 | -- | -- | > 5Mb |
|  |  | X | 101.349447 – 101.390796 | -- | -- | > 5Mb |
| 3 | PF00169 | 1 | 10.210805 – 10.381603 | -- | -- | > 5Mb |
|  |  | 2 | 38.981396 – 39.124345 | rs7578597 | 43.505684 | 4.381339 Mb down |
|  |  | 5 | 87.267888 – 87.391931 | rs12518099 | 90.250292 | 2.858361 Mb down |
|  |  | 10 | 122.374696 – 122.442602 | rs10886471 | 119.389891 | 2.984805 Mb up |
|  |  |  |  | rs10510110 | 122.432914 | inside |
|  |  | 12 | 32.399529 – 32.646050 | rs10842994 | 27.812217 | 4.587312 Mb up |
|  |  | 14 | 104.769349 – 104.795751 | rs730570 | 100.676553 | 4.092796 Mb up |
|  |  | 19 | 10.718079 – 10.833488 | -- | -- | > 5Mb |
|  |  |  | 40.230317 – 40.285536 | rs472265 | 39.090097 | 1.14022 Mb up |
|  |  | 22 | 37.697004 – 37.776556 | -- | -- | > 5Mb |
|  |  |  | 50.445000 – 50.475024 | -- | -- | > 5Mb |
|  |  | X | 101.349447 – 101.390796 | -- | -- | > 5Mb |
| 4 | PF00412 | 1 | 180.230286 – 180.278982 | -- | -- | > 5Mb |
|  |  | 9 | 136.196250 – 136.205109 | rs11787792 | 136.357696 | 0.152587 Mb down |
|  |  | 11 | 19.182030 – 19.210573 | rs5215 | 17.387083 | 1.794947 Mb up |
|  |  |  |  | rs5219 | 17.388025 | 1.794005 Mb up |
|  |  | X | 136.146702 – 136.211359 | -- | -- | > 5Mb |
| 5 | PF00413 | 11 | 102.789920 – 102.798160 | -- | -- | > 5Mb |
| 6 | PF00932 | 1 | 156.082573 – 156.140089 | -- | -- | > 5Mb |
| 7 | PF13900 | 3 | 161.083883 – 161.105384 | -- | -- | > 5Mb |
|  |  | 10 | 26.991914 – 27.100498 | -- | -- | > 5Mb |
|  |  | 12 | 50.979401 – 51.028566 | rs12304921 | 50.963759 | 0.015642 Mb up |
|  |  |  |  | rs1153188 | 54.705212 | 3.676646 Mb down |
|  |  |  | 109.573255 – 109.598117 | -- | -- | > 5Mb |
| 8 | PF00884 | 1 | 161.222292 – 161.223631 | -- | -- | > 5Mb |
|  |  | 12 | 64.713445 – 64.759447 | rs2358944 | 65.723778 | 0.964331 Mb down |
|  |  |  |  | rs1531343 | 65.781114 | 1.021667 Mb down |
|  |  |  |  | rs2261181 | 65.818538 | 1.059091 Mb down |
|  |  |  |  | rs343092 | 65.85716 | 1.097713 Mb down |
| 9 | PF01007 | 2 | 232.766464 – 232.776568 | rs1861612 | 229.657682 | 3.108782 Mb up |
|  |  | 11 | 17.385859 – 17.389331 | rs5215 | 17.387083 | inside |
|  |  |  |  | rs5219 | 17.388025 | inside |
|  |  |  | 128.891356 – 128.921035 | rs7107217 | 129.603795 | 0.68276 Mb down |
| 10 | PF00397 | 16 | 78.099413 – 79.212667 | rs17797882 | 79.373021 | 0.160354 Mb down |
|  |  |  |  | rs16955379 | 81.455768 | 2.243101 Mb down |
