# Supplementary material for: Inference of domain-disease associations from domain-protein, protein-disease and disease-disease relationships
Source: BMC Syst Biol. 2016 Jan 11;10(Suppl 1):4. doi: 10.1186/s12918-015-0247-y (PMC4895779; doi:10.1186/s12918-015-0247-y)
Supplement: Additional file 2: — Supplemental methods. This file contains a) symbols used in the equations and formulations of the methods, b) details of implementing the EM algorithm in the maximum likelihood estimation (MLE) approach, and c) details of deducing the score function in the domain-disease pair exclusion analysis (DPEA) approach. (DOCX 197 kb) [file 12918_2015_247_MOESM2_ESM.docx]

# Additional file 1: Supplemental methods

**Symbols used in the equations and formulations of the methods**

| **Symbol** | **Description** |
| --- | --- |
|  | *i*^th^ protein in the protein vector *P* |
|  | *j*^th^ disease module in the disease vector *M* |
|  | *m*^th^ domain in the domain vector *D* |
|  | *n*^th^ disease/trait in the disease/trait vector *T* |
|  | The set of associated protein-module pairs containing the domain-disease pair |
|  | The complete set of protein-module pairs containing |
| $Score(D_{m},T_{n})$ | Predicted association score between the domain-disease pair |
|  | Indicator variable denoting if domain $D_{m}$ is associated with module $T_{n}$ |
| $\psi_{ij}$ | Indicator variable denoting if protein $P_{i}$ is associated with module $M_{j}$ |
|  | false positive rate for the observed protein-module associations |
|  | false negative rate for the observed protein-module associations |
|  | Indicator variable denoting if protein $P_{i}$ and module $M_{j}$ are observed to be  associated |
| $L$ | Likelihood function for all the observed protein-module relationships |
| $\lambda_{mn}^{\text{init}}$ | Initial estimate of |
| $\vert D^{(n)}\vert$ | Number of all candidate domains for disease/trait n |
| $O$ | Set of observed protein-module relationships |
| $\Phi$ | Set of domain-disease relationships underlying every protein-module pair |
|  | Indicator variable denoting if domain $D_{m}\in P_{i}$ associates with disease $T_{n}\in M_{j}$ |
| $\left\vert X_{mn}\right\vert$ | Number of associated $D_{m},T_{n})$ domain-disease pairs in the associated  protein-module pairs |
| $\left\vert Y_{mn}\right\vert$ | Number of non-associated $D_{m},T_{n})$ domain-disease pairs in the associated  protein-module pairs |
| $Z_{mn}$ | Set of non-associated protein-module pairs containing the domain-disease pair |
| $a$, $b$ | Predefined integers used in the DPEA approach |
| $\overline{\lambda}^{mn}$ | Obtained from $\lambda$ by setting the probability of domain associated with  disease to be 0 |
| $u_{p},v_{p},u_{n},v_{n},\alpha,\beta$ | Hyper-parameters used in the Bayesian approach |
|  | Identical to $\Pr(\psi_{ij}=1)$ |
| $\Delta$ | Set of domain-disease pairs underlying all associated protein-module pairs |
| $x_{mn}$ | Variable to measure the strength of potential domain-disease association between domain and disease . |
|  | Reliability rate, the probability that a protein-module association actually exists |
| LP-score | The average obtained by including each protein-module association into  the constraints with probability and performing the linear programming  1,000 times |
|  | Number of occurrences (witnesses) for a given domain-disease pair in  each associated protein-module pair $(P_{i},M_{j})$ |
| $p\text{-value}(D_{m},T_{n})$ | Frequency of obtaining the same or higher LP-score in the 1,000 runs when the  protein-module pair containing the domain-disease pair is randomized |
| pw-score | Promiscuity versus witnesses (pw)-score to each domain-disease pair |

## Maximum Likelihood Estimation (MLE) approach

From the main text, the probability of protein $P_{i}$ associating with module $M_{j}$ is

(1)

The probability for the observed protein-module association is

(2)

Then the likelihood function is

(3)

which is a function of .

Therefore we define the complete data as , in which is the set of observed protein-module relationships, and is set of domain-disease relationships underlying every protein-module pair, where if domain associates with disease/trait in the protein-module pair $(P_{i},M_{j})$ and otherwise. We derive the forms of the EM algorithm as follows.

**E-step**:

**M-step**:

(4)

The EM algorithm is implemented as follows:

**Step 1**. Initialize parameters as , and compute by Equation (1) and by Equation (2);

**Step 2**. Update parameter by Equation (4) and compute the likelihood function *L* by Equation (3);

**Step 3**. Go to Step 2, repeat until the value of *L* is unchanged (within certain error, in this paper we use 1e-5).

## Domain-disease pair exclusion analysis (DPEA) approach

In order to deduce the score function of the DPEA approach, we define as the indicator variable denoting if domain $D_{m}\in P_{i}$ associates with disease $T_{n}\in M_{j}$. For simplicity we initialize all . In addition, we also define as the number of associated domain-disease pairs in the associated protein-module pairs, as the number of non-associated domain-disease pairs in the associated protein-module pairs, and as the set of non-associated protein-module pairs containing the domain-disease pair .

Let the initial estimate of be

The likelihood of the observed protein-module associations is estimated as

Here $a$ and $b$ are predefined integers to prevent from being exactly 0 or 1 in the case of few occurrences of pairs in the data, and thus extremely high or low can arise only from large numbers of observations pertaining to the potential association of . In our study, both $a$ and $b$ are set to be 1, as was used by Riley *et al*. [[14](#_ENREF_14)].

The likelihood is a function of , and we apply an EM algorithm to estimate . Next, instead of using $\lambda_{mn}$, we use the change in log-likelihood of observed protein-module associations as a score to measure the strength of association for the domain-disease pair , when is assumed to be not associated. The score is thus defined as

where $\overline{\lambda}^{mn}$ is obtained from $\lambda$ by setting the probability of domain associated with disease to be zero, and is also estimated by the EM algorithm.
